# Substantial Downregulation of Myogenic Transcripts in Skeletal Muscle of Atlantic Cod during the Spawning Period

**DOI:** 10.1371/journal.pone.0148374

**Published:** 2016-02-04

**Authors:** Kazue Nagasawa, Elena Sarropoulou, Vigdis Edvardsen, Jorge M. O. Fernandes

**Affiliations:** 1 Marine Genomics Group, Faculty of Biosciences and Aquaculture, Nord University, 8049 Bodø, Norway; 2 Institute of Marine Biology, Biotechnology and Aquaculture, Hellenic Centre for Marine Research, 71003 Heraklion, Greece; CNRS, UMR 9197, FRANCE

## Abstract

Gonadal maturation is an extremely energy consuming process for batch spawners and it is associated with a significant decrease in growth and seasonal deterioration in flesh quality. Our knowledge about the molecular mechanisms linking sexual maturation and muscle growth is still limited. In the present study, we performed RNA-Seq using 454 GS-FLX pyrosequencing in fast skeletal muscle sampled from two-year-old Atlantic cod (*Gadus morhua*) at representative time points throughout the reproductive cycle (August, March and May). In total, 126,937 good quality reads were obtained, with 546 nucleotide length and 52% GC content on average. RNA-Seq analysis using the CLC Genomics Workbench with the Atlantic cod reference UniGene cDNA data revealed 59,581 (46.9%) uniquely annotated reads. Pairwise comparison for expression levels identified 153 differentially expressed UniGenes between time points. Notably, we found a significant suppression of *myh13* and myofibrillar gene isoforms in fast skeletal muscle during the spawning season. This study uncovered a large number of differentially expressed genes that may be influenced by gonadal maturation, thus representing a significant contribution to our limited understanding of the molecular mechanisms regulating muscle wasting and regeneration in batch spawners during their reproductive cycle.

## Introduction

In addition to its main role in animal locomotion, fast skeletal muscle also functions as a large reservoir of protein to regulate the concentration of amino acids in circulating blood [1]. These proteins are also mobilised for gonadal protein synthesis [2]. In batch spawners, periodic gonadal maturation results in seasonal changes of muscle mass, protein content and activity of proteolytic enzymes [3, 4]. In most aquaculture fish species, the number of fast skeletal muscle fibres continuously increases from the larval phase by a process called mosaic hyperplasia until the fish reaches approximately 40% its maximum fork length; further growth is due to an increase of muscle fibre mass by hypertrophy [5].

In the wild, Atlantic cod (*Gadus morhua* L.) at the Norwegian coastal cod and northeast Arctic cod reach puberty at 3–8 and 5–10 years of age, respectively [6]. In order to fulfil the continued consumer demands despite the decline of wild stocks, there has been an effort since the 1970s to develop cod farming, mainly in Norway, Iceland, and Canada. Although aquaculture production was increasing steadily, the industry faced several bottlenecks. The major issue during the ongrowing phase is the reduction of growth rate and even weight loss caused by precocious sexual maturation [7], since under farming conditions most cod reach puberty at two years of age [8]. This translates into an extended production time and reduced profitability of the cod farming industry. Moreover, gonadal maturation is associated with poor flesh quality [9, 10], which decreases the market value of the fish. In spite of their importance, the molecular networks involved in muscle wasting associated with sexual maturation have received scant attention in fish.

RNA-Seq is a powerful approach for comprehensive profiling of transcripts and it has been used to obtain an overview of the skeletal muscle transcriptome in aquaculture species such as rainbow trout [11] and gilthead sea bream [12, 13]. A recent study by Salem et al. [14] examined changes in muscle transcript levels between diploid and triploid rainbow trout but it was limited to i) females, ii) a relatively narrow time window (two to three months prior to spawning) and iii) gene pathways related to fatty acid metabolism. The aim of the present study was to investigate global changes in the fast muscle transcriptome of Atlantic cod during a reproductive cycle, with focus on transcripts involved in myogenesis. Using the 454 GS-FLX titanium next-generation sequencing technology, we covered the representative stages of a maturation cycle, namely immature (August), mature/ ripe (March) and spent (May), as previously detailed in a sister paper [15]. Differential mRNA levels were detected by the 3'-UTR tagging method, so that each valid read reflects the copy number of transcribed mRNAs [16]. Our findings give a comprehensive view of key genes involved in homeostasis of fast skeletal muscle throughout a reproductive cycle and provide clues for understanding molecular mechanism underlying the negative impact of gonadal maturation in muscle growth in aquaculture species.

## Materials and Methods

### Fish husbandry and sampling

**Fig 1:**
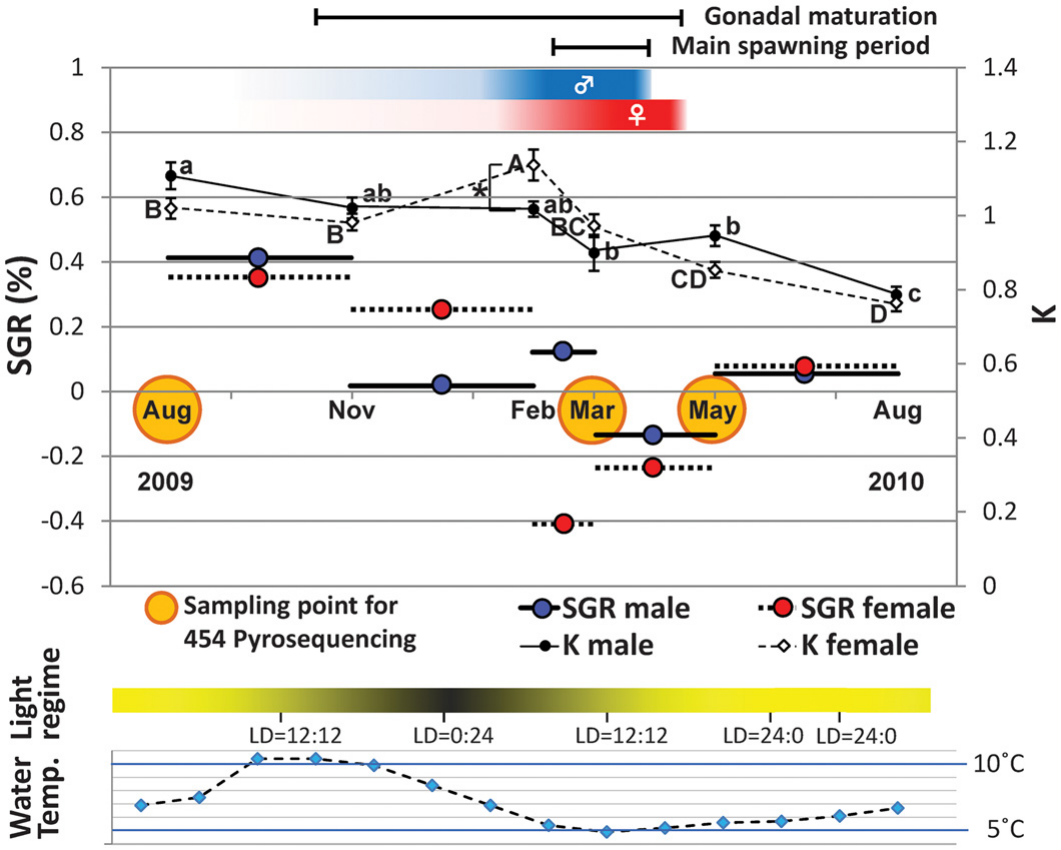
Growth history of Atlantic cod throughout their first reproductive cycle. At the start of the experiment the fish were two years old (males, n = 49; females, n = 84). Specific growth rate SGR (%) is indicated by horizontal line (males) and dotted line (females). Somatic condition factor (K) is displayed by black dots (males) and white diamonds (females). The bar underneath indicate the light regime corresponding to the natural environmental photoperiod conditions in Bodø (67°N), Norway. Sea water temperature is represented by blue diamonds with a dashed line. Asterisks (*) indicates significant differences between sexes at a particular sampling point at P < 0.05 (two-tailed t-test). Different superscript letters highlight significant differences within the same sex throughout the reproductive cycle at P < 0.05 (Tukey's multiple comparison test; lower-case letters: males; capital letters: females). Sampling points of fast skeletal muscle for 454 pyrosequencing are indicated by yellow circles.

Two-year-old Atlantic cod with an initial size of 44.8 ± 2.5 cm fork length (FL), 1.1 ± 0.2 kg total body weight (mean ± standard deviation [SD], n = 18), were reared in three land-based tanks, each holding 40 tons of sea water with approximately 100 fish at Mørkvedbukta Research Station (University of Nordland, Bodø, Norway) from July 2009 until August 2010. Sea water was pumped from 200 m depth and supplied at 7.1 ± 1.9°C (mean ± SD). Tanks were kept under a light regime corresponding to natural environmental photoperiod conditions in Bodø (67°N). A commercial diet (Amber Neptun, Skretting AS, Stavanger, Norway) was provided daily by automatic belt feeders. Approximately 10 fish from both sexes were measured for fork length and weighed in August, November 2009, February, March, May and August 2010 (Fig 1). Gutted weight (W) is calculated without liver and gonad in grams and length (L) is in centimetres. Fulton’s condition factor (K) was calculated according to Bagenal [17], while specific growth rate (SGR) was determined following Ricker [18]. Statistical differences were determined by two-way ANOVA and one-way ANOVA with subsequent Tukey’s multiple comparison tests using GraphPad Prism (GraphPad software, San Diego, USA). Normality and equal variance requirements were met.

**Fig 2:**
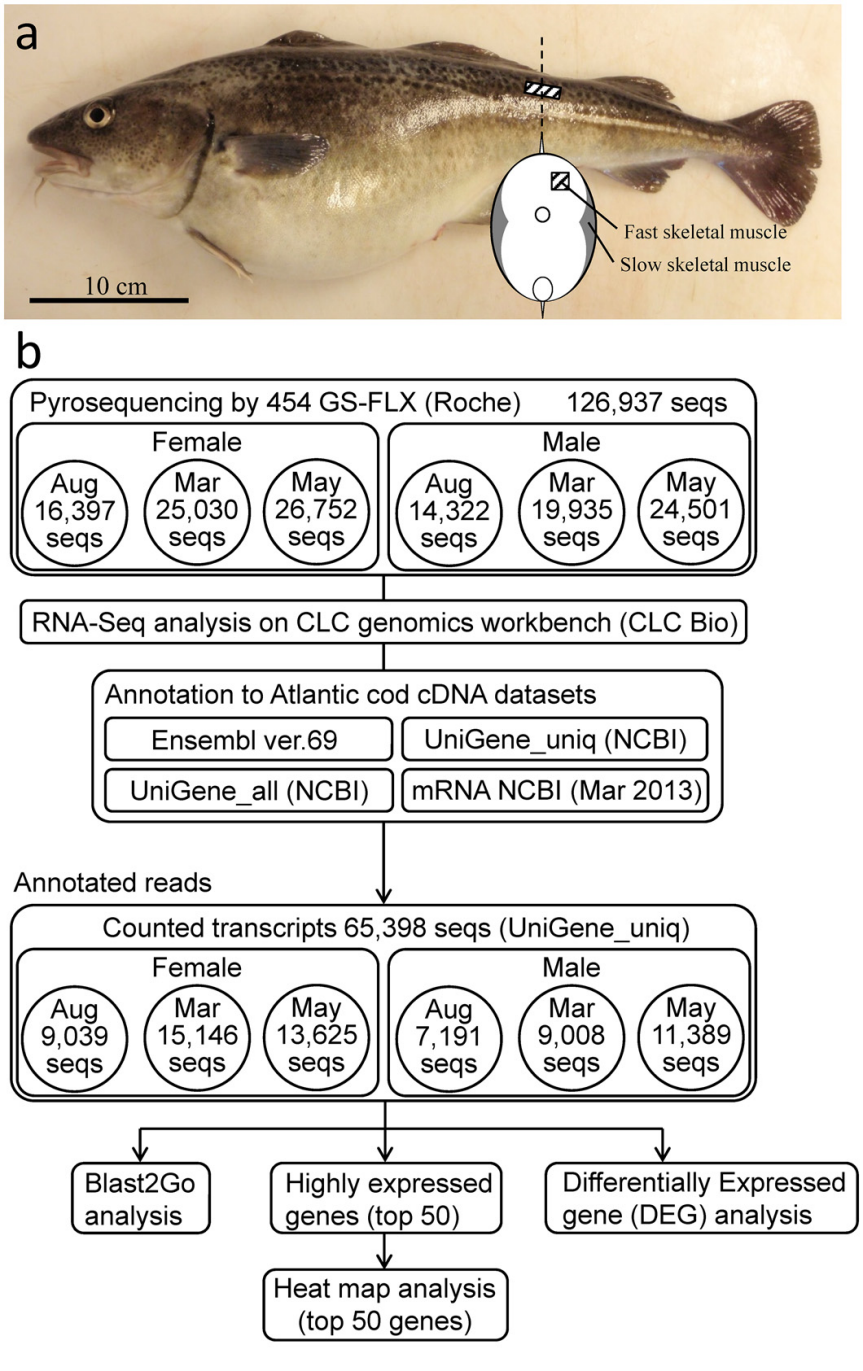
(a) Sampling positon of fast skeletal muscle and (b) schematic representation of the data processing pipeline for annotation, gene ontology, heat map, and differential expression analyses of Atlantic cod fast skeletal muscle transcriptomes for both sexes throughout the year.

For RNA extractions, 6 fish of each sex were sampled in August 2009, March and May 2010 (Fig 1). Fish were humanely killed by immersion in seawater containing 0.5 g·L^-1^ tricaine methanesulfonate (Sigma, Oslo, Norway) and fast skeletal muscle was carefully dissected from the region posterior to the second dorsal fin, taking special care to avoid skin and red muscle, as detailed in Fig 2a. Sampled muscle tissues were snap-frozen in liquid nitrogen and stored at -80°C until RNA extraction. All procedures were conducted in accordance to the guidelines set by the National Animal Research Authority (Forsøksdyrutvalget, Norway).

### RNA extraction and 454 pyrosequencing

Total RNA from fast skeletal muscle was individually extracted from 36 fish (six males and six females in three sampling points) using the *mir*Vana^™^ miRNA isolation kit (Life Technologies, Paisley, UK), according to the manufacturer’s instructions. RNA integrity and quantity were determined using the Agilent 2100Bioanalyzer (6000 Nano LabChip, Agilent Technologies, CA, USA). For cDNA library construction, total RNA from six fish of the same sex at each sampling point were pooled and used for a modified version of the 454 cDNA rapid library preparation [16]. In brief, 2 μg total RNA was fragmented by adding 2 μl fragmentation solution in a total volume of 18 μl, vortexed and incubated at 72°C for 30 s. Precipitation was performed at -20°C overnight in a total volume of 500 μl with 50 μl NaOAc, 2.5 ml EtOH and 1 μl glycogen (Life Technologies). Synthesis of first strand cDNA was performed using the oligo nucleotide primer 5’-TTTTTTCTTGTTTTCTTTTCTTV-3’ according to the cDNA library protocol of GS-FLX454. Quantification of the library was assessed by the Quantiflur ST Fluorometer (SB Biotechnology Suppliers S.A, Athens, Greece) and fragment size distribution was determined on the Bioanalyzer. Next-generation sequencing was performed according to GS-FLX Titanium protocols. All sequencing data were submitted to the NCBI SRA database (female skeletal muscle in August; SRR955389, March; SRR955390, May; SRR955394, male skeletal muscle in August; SRR955391; March; SRR955392, May; SRR955396).

### Fragment annotation

All sff files were converted to fasta files, and raw sequence reads were trimmed by SeqClean (http://sourceforge.net/projects/seqclean/files/). The fasta file containing cleaned sequences per library was mapped to the *G*. *morhua* reference transcriptome data using the RNA-Seq analysis module in CLC Genomics Workbench (version 5.5.1, CLC bio, Aarhus, Denmark). In order to validate the most appropriate reference cDNA data set for fragment mapping, four different reference transcriptomes were evaluated: i) *G*. *morhua* Ensembl cDNA data (22,618 sequences) containing known, novel and pseudogene sequences downloaded from Ensembl (ftp://ftp.ensembl.org/pub/release-69/fasta/gadus_morhua/cdna/), ii) *G*. *morhua* UniGene_uniq (41,275 sequences), iii) *G*. *morhua* UniGene_all (236,631 sequences, http://www.ncbi.nlm.nih.gov/unigene/), and iv) *G*. *morhua* mRNA_NCBI (42,058 sequences, http://www.ncbi.nlm.nih.gov/nuccore). After validation, fragments mapped to the reference cDNA data were extracted and used for functional gene annotation (gene ontology; GO, www.geneontology.org) using Blast2GO-PRO v. 2.6.4 [19] with default annotation parameters (BLAST step: Blast program, blastx; Blast DB, nr; Blast E-value, 1.0E-3. Annotation step: E-value hit filter, 1.0E-6; Annotation threshold, 55; GO weight, 5).

### RNA-seq data analysis

Gene expression levels were normalised as reads per kilobase of exon model per million mapped reads (RPKM) using the CLC transcriptomic analysis module. The top 50 highly expressed UniGene or Ensembl genes selected by the sum of RPKM values in all libraries (RPKM threshold in UniGene; > 2,129 or RPKM threshold in Ensembl; > 4,389) were used for heat map analysis with hierarchical clustering using PermutMatrix (http://www.lirmm.fr/~caraux/PermutMatrix/EN/). The Excel statistical function CHISQ.TEST was used to obtain the p-values for differences between RPKM values within sex. Theoretical values were obtained from means of RPKM values for each sex.

Differentially expressed genes (DEGs), up- or downregulated between sampling points, were defined when their log_2_ fold change was > 2 or < -2, respectively (P < 0.001) [20]. The scatter plot for DEGs with log_2_ Ratio was generated by Tableau 8.0 (tableausoftware.com).

### Phylogenetic analysis

A phylogeny of teleost myosin heavy chain (*myh*) genes was generated with Atlantic cod *myh* paralogues identified in genomic and transcriptomic data (S5 Table), teleost *myh13* genes from Ensembl (release 71, S6 Table) and additional zebrafish *myh* genes obtained from NCBI (S7 Table). Deduced amino acid sequences were aligned with MUSCLE (drive5.com) and aligned sequences were used for Bayesian phylogenetic analysis (MrBayes v3.1.2, mrbayes.csit.fsu.edu), as detailed elsewhere [21].

### Quantitative real-time PCR (qPCR)

Total fast skeletal muscle RNA and cDNA was obtained from six fish of each sex in August and November 2009, and in February, March and May 2010 from the same population used for RNA-Seq above. Quantification of transcript levels of five DEGs and three reference genes was performed by qPCR using gene specific primer sets (S8 Table), as previously described [21]. Validation of acidic ribosomal protein (*arp*), eukaryotic elongation factor 1a (*eef1a*) and ubiquitin (*ubi*) as reference genes for this experimental setup was investigated and raw expression data were normalized with geNorm (medgen. ugent.be/,jvdesomp/genorm/) normalization factors corresponding to the geometric average of *arp* and *ubi*, which were found to be the two most stable genes. Differences of relative mRNA levels among seasons were examined by Mann-Whitney tests, since the data were not normally distributed. For comparisons within sex, a Kruskal-Wallis non-parametric ANOVA was used, followed by a Dunn’s post-hoc test for multiple comparisons. For all statistical tests, significance levels were set at p < 0.05.

## Results

### Fish growth throughout the annual reproductive cycle

In order to detect changes of the transcriptome profiles in fast skeletal muscle throughout the annual reproductive cycle, we kept two-year-old Atlantic cod in land-based tanks from July 2009 to August 2010. Spermiation was seen in some males in February and March, whereas ovulation was seen in several females in May, indicating that the fish in this study displayed a similar reproductive cycle as wild cod. Their spawning period ranged from February to early May. Further details of their reproductive status with histological observations at each sampling point can be found in our sister paper [15]. These data clearly showed that the fish were at the following maturation stages: start of the maturation process in August, ripe in March and spent in May. The initial mass of gutted fish did not differ between males and females in August 2009 (P = 0.53, n = 18) and there were no significant differences between sexes for gutted weight throughout the experiment (November 2009; P = 0.97, n = 23, February 2010; P = 0.09, n = 24, March 2010; P = 0.48, n = 25, May 2010; P = 0.82, n = 21, August 2010; P = 0.64, n = 22).

In contrast, there were changes in specific growth rates (SGR) throughout the reproductive cycle (Fig 1). Between August and November 2009, both males and females showed the highest SGR at 0.41 and 0.35%, respectively. A decline of male SGR was detected in November 2009 to February 2010 (0.02%) and then from March to May 2010 (-0.13%). A remarkable decrease of SGR in females was detected in February to March 2010 (-0.41%), continuing at negative values from March to May 2010 (-0.23%). After the spawning period, a recovery in SGR was detected for both males (0.05%) and females (0.08%), from May to August 2010. The Fulton’s condition factor (K) in females was significantly elevated from November 2009 (K = 0.98) to February 2010 (K = 1.14) and gradually decreased until August 2010 (K = 0.76), whereas the K in males showed a decrease from February (K = 1.02) to March 2010 (K = 0.90) and from May (K = 0.95) to August (K = 0.79) (Fig 1). A significant difference in K between sexes was only found in February 2010 (P < 0.05, male; n = 8, female; n = 16).

### 454 pyrosequencing and RNA-Seq analysis

Six cDNA libraries were constructed from fast skeletal muscle from both female and male cod at three different points of the reproductive cycle: August (before entering oocyte maturation/spermiogenesis), March (spawning/spermiation) and May (after spawning/spermiation) as previously detailed in a sister paper [20]. Pyrosequencing of the above six cDNA libraries yielded a total of 126,937 valid sequence reads (Fig 2b) with an average length of 546 nucleotides and 52% GC%. In female fast skeletal muscle cDNA libraries, 16,397, 25,030 and 26,752 valid reads were obtained from the August, March and May samples, respectively. A total of 14,322, 19,935 and 24,501 valid reads were generated in August, March and May male muscle cDNA libraries, respectively.

Prior to annotation and RNA-Seq analysis, an evaluation of the most suitable reference transcriptome was performed by annotating all sequenced valid reads in this experiment (126,937 sequences) using four different reference cDNA data sets (S1a Fig). Amongst the annotation results, 46.9% (59,581 sequences) of all valid reads were uniquely annotated against UniGene_uniq, whereas only 16.6% (21,240 sequences) were uniquely annotated to Ensembl cDNA data set (S1b Fig). A considerable number of reads were annotated to more than one sequence contigs of the reference data sets UniGene_all and mRNA_NCBI at 39,456 (31.1%) and 27,291 (21.5%), respectively (S1b Fig), suggesting that these two reference sets might contain relatively high number of very similar sequence entries. Therefore, the UniGene_uniq data set was chosen as the best reference transcriptome. Meanwhile, the Ensembl cDNA collection was also chosen as a complementary reference data set, since it contains novel or unknown transcript sequences predicted *in silico* and which may not be present in the UniGene_Uniq data. In particular, 8,080 to 13,847 (40.5 to 55.3%) and 2,824 to 5,076 (11.3 to 31.0%) valid reads per library were uniquely annotated against UniGene_uniq (Table 1a) and Ensembl cDNA data (Table 1b), respectively. Reads per kilobase of exon model per million mapped reads (RPKM) values of all six libraries ranged from 0 up to 182,290 in UniGene_uniq (Table 2) or up to 96,472 in Ensembl for all six libraries (S1 Table).

**Table 1:** Number of total fragment for six adult Atlantic cod fast skeletal muscle 454 GS-FLX cDNA libraries and annotated fragment against UniGene or Ensembl reference cDNA data sets according to month and sex.

**a. UniGene**							
UniGene	Female			Male			Total
41,275 sequences	Aug	Mar	May	Aug	Mar	May	
Total fragments	16,397	25,030	26,752	14,322	19,935	24,501	126,937
Counted fragments	9,039	15,146	13,625	7,191	9,008	11,389	65,398
uniquely	8,317	13,847	12,466	6,514	8,080	10,357	59,581
non-specifically	722	1,299	1,159	677	928	1,032	5,817
Unacounted fragments	7,358	9,884	13,127	7,131	10,927	13,112	61,539
Annotated fragments (%) (Uniquely/total)	50.7	55.3	46.6	45.5	40.5	42.3	46.9
Matched cDNA in UniGene cDNA	672	1,353	1,142	660	1,019	864	3,059
**b. Ensembl**							
UniGene	Female			Male			Total
22,618 sequences	Aug	Mar	May	Aug	Mar	May	
Total fragments	16,397	25,030	26,752	14,322	19,935	24,501	126,937
Counted fragments	5,112	2,837	5,295	2,786	2,601	4,353	22,984
uniquely	5,076	2,824	5,274	2,771	2,591	4,338	21,024
non-specifically	36	13	21	15	10	15	86
Unacounted fragments	11,285	22,193	21,457	11,536	17,334	20,148	103,953
Annotated fragments (%) (Uniquely/total)	31.0	11.3	19.7	19.3	13.0	17.7	16.6
Matched cDNA in UniGene cDNA	394	675	559	313	462	398	1,499

**Table 2:** Top 50 highly expressed transcripts according to combined RPKM values from all libraries.

	Seq. Name	Range	p-value Female	p-value Male	Seq. Description	Hit ACC	E-value	Similarity (%)
1	gnl|UG|Gmr_S60871749	182290	0	0	hypothetical protein OXYTRI_13059	EJY66654	1.12E-14	77
2	gnl|UG|Gmr_S55464446	79514	0	0	chk1 checkpoint-like partial	AEM37715	4.57E-32	77
3	gnl|UG|Gmr_S55460252	62267	0	0	alpha cardiac-like isoform 2	BAG51757	2.83E-115	99
4	gnl|UG|Gmr_S55464884	51778	0	0	hypothetical protein	XP_001916399	4.00E-31	85
5	gnl|UG|Gmr_S60847006	40385	0	0	myosin heavy chain	BAA33452	2.12E-133	93
6	gnl|UG|Gmr_S60854098	35641	0	0	novel sal-like protein	AEM37715	4.29E-26	73
7	gnl|UG|Gmr_S55473677	28186	0	0	myosin heavy chain	BAA19070	0	96
8	gnl|UG|Gmr_S60850998	26326	0	4.5E-235	alpha cardiac muscle 1	AAM21702	0	100
9	gnl|UG|Gmr_S60867323	20318	0	0	myosin light chain 2	BAB18578	1.73E-111	96
10	gnl|UG|Gmr_S44201072	18051	0	0	heavy polypeptide skeletal muscle	XP_003976385	2.39E-115	96
11	gnl|UG|Gmr_S44200679	15406	0	0	alpha skeletal muscle	AAA29846	4.09E-51	100
12	gnl|UG|Gmr_S60849470	13635	0	0	parvalbumin beta	Q90YK9	2.49E-65	90
13	gnl|UG|Gmr_S60846140	12150	0	0	myosin heavy chain	BAA33452	1.64E-102	92
14	gnl|UG|Gmr_S41411552	12023	0	3.7E-228	myosin heavy chain	BAA12887	1.40E-168	90
15	gnl|UG|Gmr_S41411187	10982	0	0	myosin heavy chain	BAA19070	1.59E-128	96
16	gnl|UG|Gmr_S41410886	10406	0	0	heavy polypeptide skeletal muscle	AAI55231	3.49E-156	93
17	gnl|UG|Gmr_S41438721	9075	0	1.7E-239	troponin fast skeletal muscle-like	AAM16155	4.41E-102	84
18	gnl|UG|Gmr_S55468166	8550	0	0	parvalbumin beta	AAK63086	9.61E-49	94
19	gnl|UG|Gmr_S60841836	8304	1.0E-152	1.3E-04	beta-actin	ABD65243	0	99
20	gnl|UG|Gmr_S60870650	7307	1.2E-252	0	---NA---			
21	gnl|UG|Gmr_S60842126	6841	0	2.1E-276	myosin heavy chain	BAG16353	0	96
22	gnl|UG|Gmr_S60864027	6654	0	0	alpha cardiac muscle 1	NP_001098276	0	100
23	gnl|UG|Gmr_S59565731	6595	0	2.1E-85	myosin heavy chain	AEA36763	5.17E-109	99
24	gnl|UG|Gmr_S60859527	6515	1.3E-114	0	rrna promoter binding protein	AAS66225	1.65E-64	85
25	gnl|UG|Gmr_S60862414	6266	0	0	fast white muscle troponin t embryonic isoform	AAM21701	2.11E-69	92
26	gnl|UG|Gmr_S60864365	6204	0	0	myosin light chain 3	BAB18577	8.67E-102	93
27	gnl|UG|Gmr_S55460118	5662	0	0	40s ribosomal protein s3a	A2Q0R8	0	98
28	gnl|UG|Gmr_S60845540	5566	1.0E-59	0	GAPDH	AAL05892	0	96
29	gnl|UG|Gmr_S60871748	4792	0	0	syntaxin binding protein 1	XP_004075137	0	94
30	gnl|UG|Gmr_S55461537	4420	4.7E-20	2.6E-231	---NA---			
31	gnl|UG|Gmr_S60869000	4273	0	0	beta-enolase	XP_004085855	0	96
32	gnl|UG|Gmr_S55469074	4273	0	0	hypothetical protein	AAX30301	1.83E-27	92
33	gnl|UG|Gmr_S55478779	3852	0	1.7E-243	amp deaminase 1	NP_957187	4.55E-132	91
34	gnl|UG|Gmr_S60842128	3714	9.5E-207	0	myosin heavy chain	BAF75963	2.95E-54	95
35	gnl|UG|Gmr_S60835498	3557	2.4E-290	0	nebulin	CAG08263	2.35E-72	81
36	gnl|UG|Gmr_S60842632	3420	0	1.4E-246	troponin skeletal muscle	XP_003978216	5.62E-85	93
37	gnl|UG|Gmr_S41357853	3182	2.8E-249	0	titin a	ABG48500	2.00E-97	87
38	gnl|UG|Gmr_S41427575	3130	5.2E-174	4.8E-244	titin	XP_003458433	8.00E-79	94
39	gnl|UG|Gmr_S55463693	3079	0	0	ribosomal protein l7	BAF98652	4.94E-48	85
40	gnl|UG|Gmr_S60849377	2935	0	9.9E-86	cyclin g1	AFU80860	5.15E-165	83
41	gnl|UG|Gmr_S41426120	2906	1.6E-253	9.6E-223	titin	XP_003458433	4.00E-140	92
42	gnl|UG|Gmr_S41427432	2803	0	2.3E-37	titin	XP_003458433	3.00E-128	90
43	gnl|UG|Gmr_S60843589	2693	3.7E-212	0	---NA---			
44	gnl|UG|Gmr_S60867290	2506	0	7.7E-132	myosin light chain 1	BAA95143	2.13E-108	93
45	gnl|UG|Gmr_S55459862	2506	1.6E-68	2.4E-59	nucleoside diphosphate kinase b	XP_003442598	5.01E-48	84
46	gnl|UG|Gmr_S60853584	2461	2.7E-59	8.6E-259	neurobeachin	XP_003458317	5.49E-52	98
47	gnl|UG|Gmr_S60867017	2440	0	2.4E-10	titin a	ABG48500	8.00E-108	87
48	gnl|UG|Gmr_S60857401	2377	0	0	calsequestrin-1 precursor	XP_003457488	7.02E-64	90
49	gnl|UG|Gmr_S60843077	2165	2.4E-121	2.1E-145	SERCA 1-like	P70083	0	91
50	gnl|UG|Gmr_S44167007	2129	2.1E-184	3.7E-206	---NA---			

Range value was obtained from sum of RPKM value of all libraries. p-value indicate that the probability whether the observed RPKM difference among libraries is significant for each UniGene. Sequence description, hit NCBI accession number, e-value, and similarity were obtained by blastx against the NCBI nr database.

### GO functional annotation

**Fig 3:**
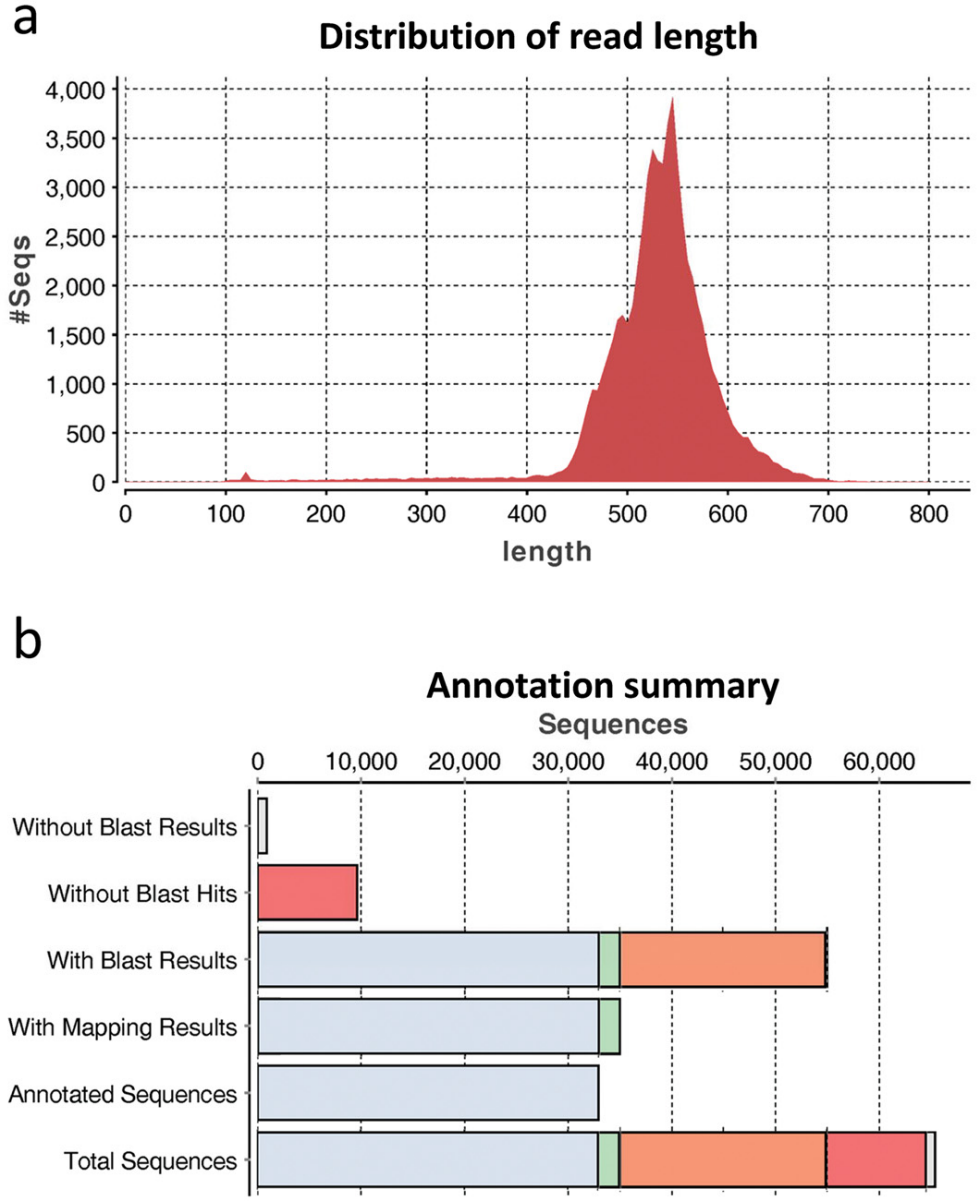
Overview of functional annotation results of Atlantic cod fast skeletal muscle transcriptome. Analysis was performed by Blast2GO-PRO software. a) Distribution of annotated sequence reads to UniGene cDNA reference data. b) Functional annotation results of Atlantic cod fast skeletal muscle transcriptome annotated to UniGene cDNA reference data.

**Fig 4:**
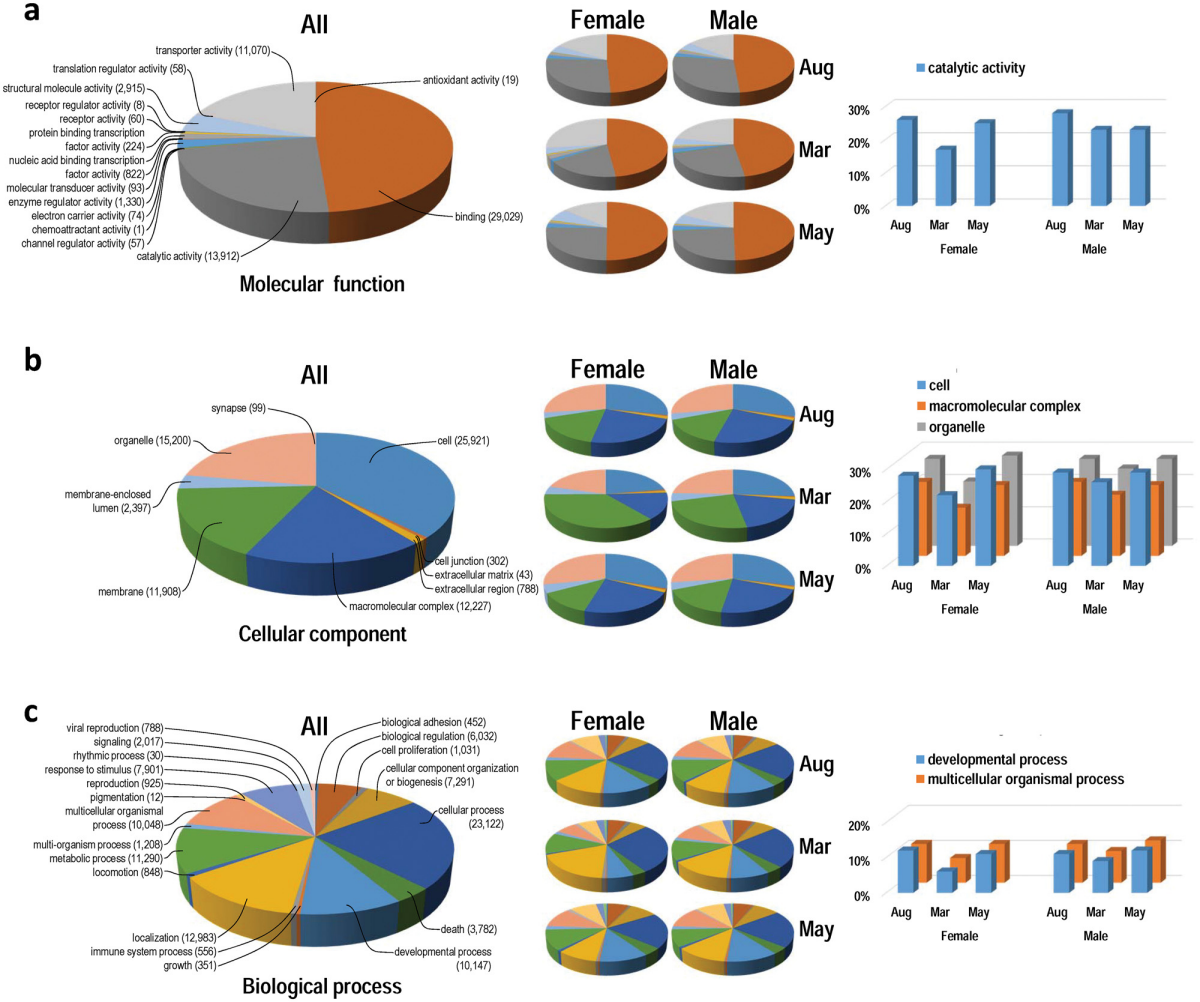
Gene ontology (GO) analysis of Atlantic cod fast skeletal muscle transcriptome annotated to UniGene cDNA reference data. Analysis was performed with Blast2GO-PRO. a) Distribution of transcripts with major category of molecular functions at level 2. b) Distribution of transcripts with major category of cellular component. c) Distribution of transcripts with major category of biological process. The larger pie charts display the combined transcript data from six libraries, whereas smaller pie charts indicate individual data for each library.

FASTA sequences of uniquely annotated fragments to UniGene data were extracted and individually submitted to Blast2GO analysis. Amongst the six libraries, 7,503 to 13,338 sequences (83.3 to 88.1%) had blastx hits against the NCBI non-redundant (nr) database (S2 Table). Within the assigned sequences for each library, 47.9 to 57.8% were mapped and then 44.8 to 55.7% were eventually annotated with GO terms (S2 Table). A brief overview of combined sequence reads of all libraries is found in S2 Table and Fig 3a and 3b. Fig 4 shows the fraction of reads assigned to various GO biological processes (set seq filter; 10 and node score filter; 10), cellular component and molecular function at level 2. The distribution of GO functions revealed that a higher proportion of genes whose products were involved in binding (48.6%), catalytic (23.3%) and transporter activity (18.6%) was identified in the category of molecular functions of all combined data from the six libraries (Fig 4a). A significant number of cellular component GO terms were associated with cell (37.6%), organelle (22.1%), macromolecular complex (17.7%) and membrane (17.3%) components (Fig 4b). Within biological processes, cellular process (22.9%), localisation (12.9%), metabolic process (11.2%), developmental process (10.1%) and multicellular organismal process (10.0%) were highly represented (Fig 4c). The direct GO count showing specific GO terms for all annotated fragments from six libraries indicated that a number of sarcomere components could be detected in the cellular component annotation (S2a–S2c Fig). The compositions of GO did not differ significantly amongst six libraries in molecular function, cellular component or biological process (Fig 4). However, a considerable decrease in proportion of several components/ processes was found in March for both sexes (Fig 4, S3 Table): namely catalytic activity in molecular function; cell, macromolecular complex and organelle in cellular component; developmental process and multicellular organismal in biological process.

### Heat map analysis and expression analysis for highly expressed genes

The top 50 highly expressed UniGenes among the 3,059 identified in all six libraries were sorted by range values (Table 2). These top 50 entries comprised mainly myofibrillar genes: actin, alpha cardiac muscle (*actc*), actin, alpha skeletal muscle (*acta*), β-actin (*actb*), β-enolase (*eno3*), calsequestrin (*casq*), myosin heavy chain (*myh*), myosin light chain (*myl*), nebulin (*neb*), parvalbumin (*pvalb*), sarcoplasmic endoplasmic reticulum calcium atpase 1-like (*atp2a*), titin (*ttn*) and troponin skeletal muscle t (*tnnt*). Some ribosomal protein genes and housekeeping genes (e.g., glyceraldehyde-3-phosphate dehydrogenase (*gapdh*), *actb*) were also in this list. Additional myofibrillar genes were identified in the top 51–100 highly expressed UniGenes: creatine kinase M-type (*ckm*), enolase 1 (*eno1*), fibulin-7-like (*fbln7*), lactate dehydrogenase-a (*ldha*), myomesin-1 (*myom1*), myosin binding protein h (*mybph*), myozenin-1 (*myoz1*), reticulon-2-like (*rtn2*), triosephosphate isomerase (*tpi*), tropomodulin 4 (*tmod4*) and tropomyosin (*tpm*) (data not shown).

**Fig 5:**
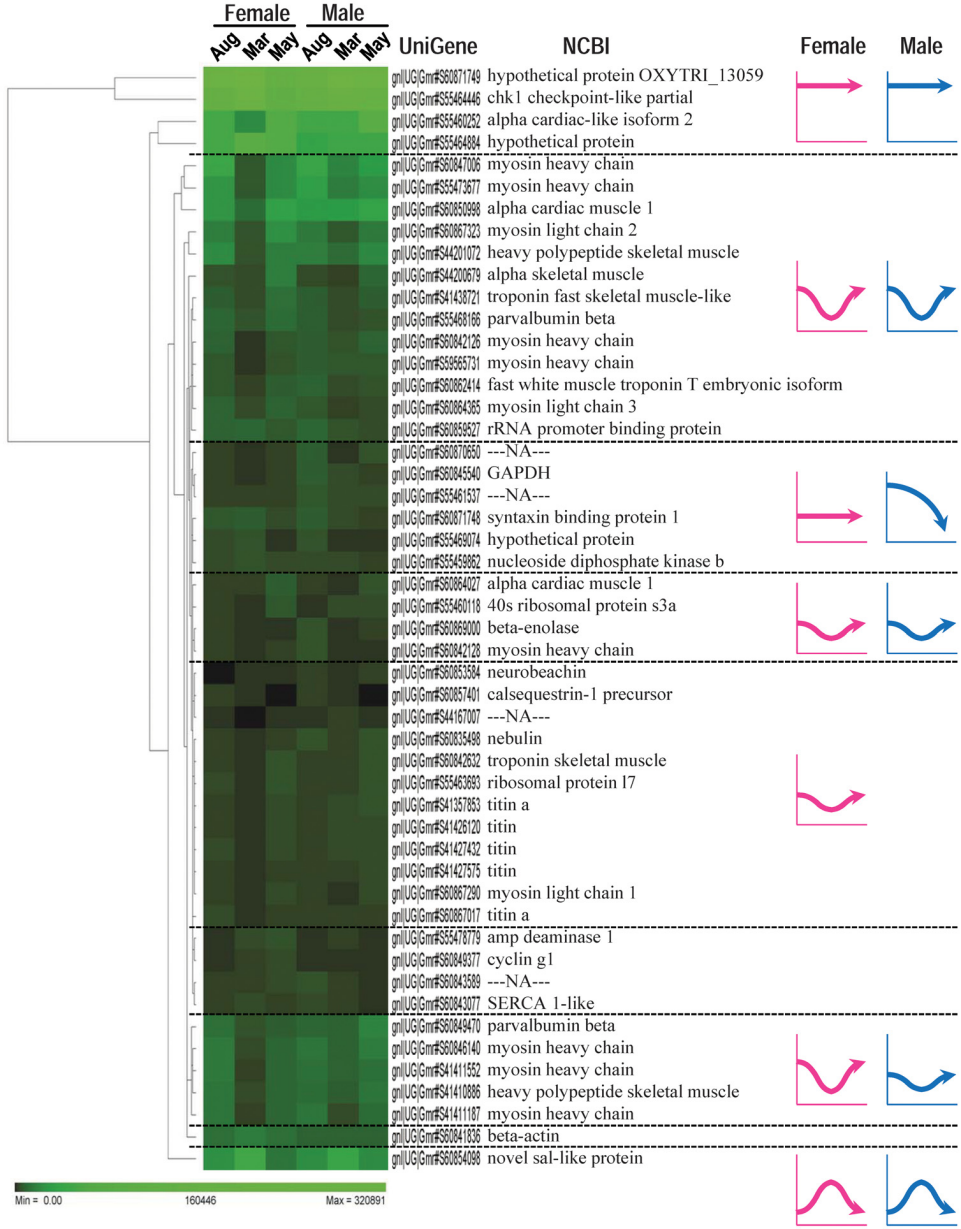
Heat map of the top 50 highly expressed genes. The map contains 50 rows and 6 columns corresponding to the top 50 highly expressed genes and sampling points for both sexes, respectively. All 50 genes have individual UniGene accession numbers and their common gene name registered in NCBI. Several UniGenes (e.g., myosin heavy chain and titin) share the same common gene name but are referred to as individual isoforms, as long as they have unique UniGene accession numbers. Typical clusters containing genes that show similar expression patterns are separated by a dashed line and their expression patterns throughout the year are summarised with arrow graphs for both females (pink arrows) and males (blue arrows).

Hierarchal clustering of the top 50 UniGenes revealed several clusters with different expression patterns throughout the year for either sex (Fig 5). β-actin (gnl|UG|Gmr_S60841836) was expressed at a stable level at all time points, implying that RPKM values were not biased by 454 pyrosequencing or annotation amongst six libraries. Two isolated clusters containing 1^st^ to 4^th^ highest expressing UniGenes such as chk1 checkpoint-like (gnl|UG|Gmr#S55464446) and hypothetical protein (gnl|UG|GmrS55464884) displayed constant expression at a high level in all sampling points. RPKM values for the novel sal-like protein (*sall*) (gnl|UG|Gmr_S60854098) had a peak in March for both sexes, in contrast to the clusters comprising myofibrillar genes. Notably, the cluster including four *myh* UniGenes (gnl|UG|Gmr_S60847006, gnl|UG|Gmr_S55473677, gnl|UG|Gmr_S60842126, gnl|UG|Gmr_S59565731) and two *myl* UniGenes (gnl|UG|Gmr_S60867323, gnl|UG|Gmr_S60864365) showed a remarkable decrease in March for both sexes. Another cluster composed of three *myh* UniGenes (gnl|UG|Gmr_S60846140, gnl|UG|Gmr_S41411552, gnl|UG|Gmr_S41411187) and *pvalb* (gnl|UG|Gmr_S60849470) displayed a decrease in March, which was more pronounced in female than in males. Meanwhile the cluster containing five *ttn* UniGenes (gnl|UG|Gmr_S41357853, gnl|UG|Gmr_S41426120, gnl|UG|Gmr_S41427432, gnl|UG|Gmr_S41427575, gnl|UG|Gmr_S60867017) decreased in females but not in males in March. A heat map of the top 50 highly expressed Ensembl genes (S3 Fig, S1 Table) also showed that the RPKM value for *myh* and *myl* Ensembl genes decreased in March for both sexes.

### Differentially expressed genes (DEGs) with seasonal changes within the same sex

**Fig 6:**
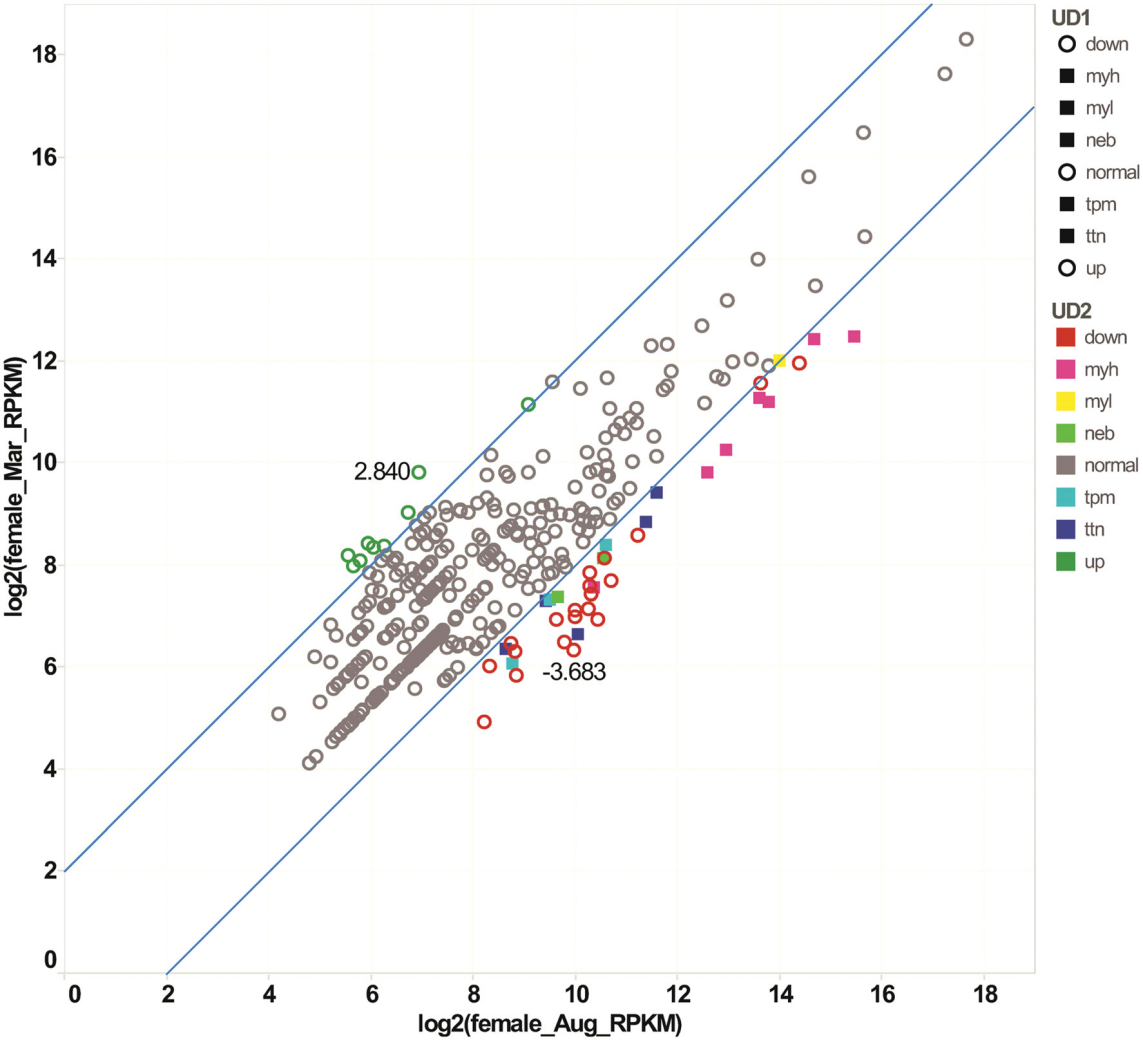
Differentially expressed genes (DEGs) in fast skeletal muscle of female Atlantic cod between August and March. DEGs were determined by pairwise comparison when their log_2_ fold change was log_2_ Ratio > 2 (upregulated gene) or log_2_ Ratio < -2 (downregulated gene). X- and y-axis indicate the log_2_ (RPKM values of female muscle in August) and log_2_ (RPKM values of female muscle in March), respectively. Green circles represent upregulated DEGs and red circles indicate downregulated DEGs. Grey circles show the UniGenes that did not display significant changes.

To identify differentially expressed genes (DEGs), log_2_ fold changes in RKPM value between sampling points were calculated for all 3,059 UniGenes identified in the six libraries. In total, 153 UniGenes were considered as DEGs displaying significant downregulation and/or upregulation for either sex throughout the year (S4 Table). In March compared to August, 38 genes in females and 23 genes in males were significantly downregulated whereas 10 genes in females and eight genes in males were significantly upregulated (Table 3). Between May and March, 15 genes in females and 12 genes in males were significantly downregulated whereas 59 genes in females and 11 genes in males were significantly upregulated. It is noteworthy that the DEGs were mainly composed of highly expressed myofibrillar genes (Fig 5) and several ribosomal proteins. In particular, a considerable number of *myh* UniGenes (eight paralogues), *tpm* (four genes), *neb* (five genes) and *ttn* (13 genes) were found (S4 Table). Remarkably, the scatter plot for females in March compared to August revealed a significant decrease of seven *myh* UniGenes, one *myl* UniGene and several UniGenes of myofibrillar genes (Fig 6). Scatter plots for each pairwise comparison revealed additional down- and upregulated DEGs (S4a–S4f Fig).

**Table 3:** Overview of differentially expressed genes (DEGs) in fast skeletal muscle of Atlantic cod compared by expression level (RPKM) between the libraries. Significantly upregulated or downregulated genes are selected by the set values log_2_ Ratio > 2 and log_2_ Ratio < -2, respectively.

	Female			Male		
	Mar/Aug	May/Mar	May/Aug	Mar/Aug	May/Mar	May/Aug
Upregulated	10	59	26	8	11	6
Downregulated	38	15	9	23	12	13

### Myosin heavy chain in Atlantic cod

**Fig 7:**
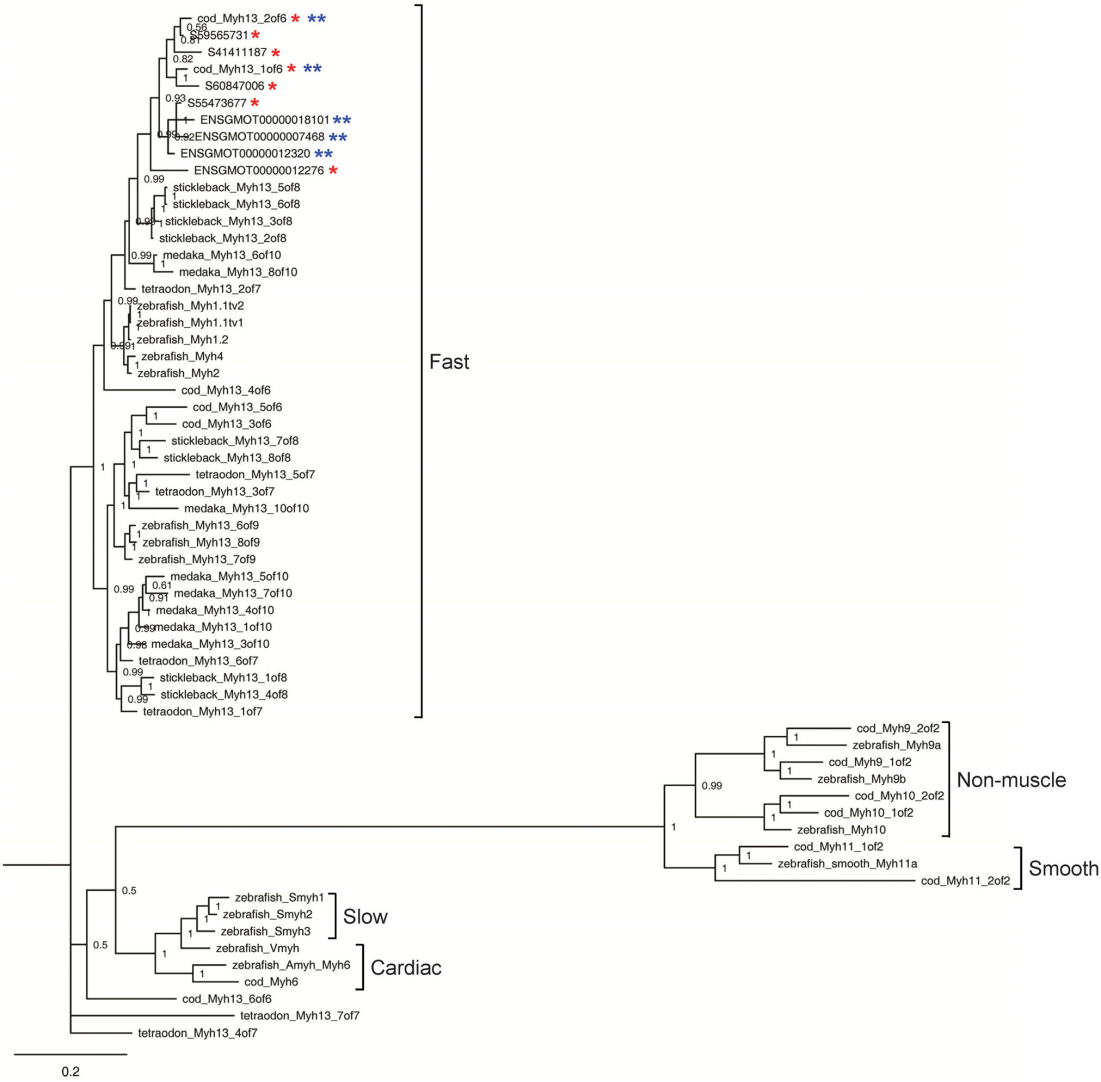
Phylogenetic inference of myosin heavy chain (*myh*) genes in vertebrates. Red and blue asterisks indicated the *myh* clones annotated to UniGene and Ensembl reference data sets, respectively. Numbers at the nodes indicate posterior probability values obtained from the Bayesian analysis. Ensembl transcript IDs and NCBI accession nos. of teleost *myh13* genes can be found in S6 and S7 Tables, respectively.

All available *myh* genes (*myh6*, two *myh9*, two *myh10*, two *myh11*, *myh12*, six *myh13* and two *myh14*) were *in silico* cloned from the Atlantic cod genome data in Ensembl (S5a Table). Several *myh* isoforms for five Ensembl clones (S5b Table) and nine UniGenes (S5c Table) were also detected. Further BLAST-based analysis revealed that three Ensembl and five UniGene entries may correspond to novel *myh* isoforms (S5b and S5c Table). Bayesian phylogenetic reconstruction of the teleost *myh* gene families is shown in Fig 7. The topology of the phylogenetic tree indicated that all eight putative *myh* isoforms and two Atlantic cod *myh13* isoforms (1 of 6 and 2 of 6) identified in this study belonged to a same clade, which is a fast muscle type. Other Atlantic cod *myh* paralogues found in Ensembl clustered with the expected zebrafish *myh* homologues, including other types of *myh* such as slow, cardiac and smooth muscle types.

### qPCR verification of five DEGs

**Fig 8:**
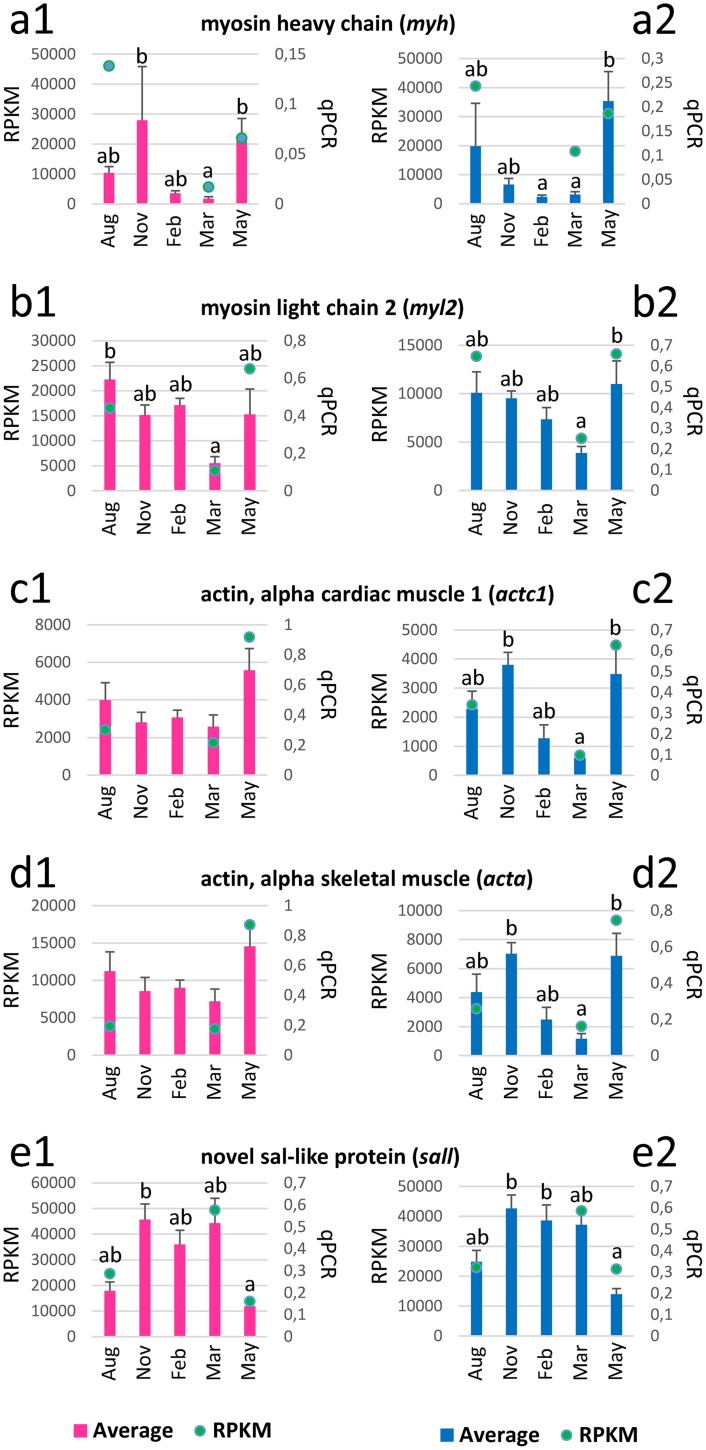
qPCR quantification of five DEGs in fast skeletal muscle throughout a reproductive cycle and comparison with RPKM values from RNA-Seq. Pink and blue bars show the relative mRNA expression in females and males, respectively. Green dots indicate RPKM values from the RNA-Seq. Error bars show S.E.M. at each point (n = 6 for each sex). Different superscript letters indicate significant differences within sex at p < 0.05.

Five genes were selected from the DEGs that were expressed at relatively high levels with prominent seasonal changes in the above DEG analysis based on RPKM values. In brief, four DEGs (i.e., *mhc*, *myl2*, *actc1*, and *acta*) that are myofibrillar genes showed substantial and significant decrease during the spawning period (i.e., February and March) in females and/or males (Fig 8a–8d). In contrast, novel *sall* transcript levels increased during the spawning period. Correlation analysis indicated that qPCR data for the five DEGs were well correlated with the RPKM results from 454 pyrosequencing (S5 Fig).

## Discussion

Similarly to other batch spawners, Atlantic cod shows a large increase of gonad weight during the reproductive season [15], indicating a significant seasonal shift of energy metabolism. This represents a major bottleneck for fish farmers, since gonadal maturation often results in a decrease in fish muscle yield, slowdown of growth rate and undesirable extension of the rearing period [22, 23]. Accordingly, we observed a dramatic decrease in female and male specific growth rates in February to March and November to February, respectively, and a prominent reduction in condition factor for both sexes in March. To the best of our knowledge, this is the first transcriptomics report of changes in expression levels of multiple myosin heavy chain 13 (*myh13*) and other key genes related to myogenesis in muscle of a batch spawner teleost during a reproductive cycle.

Among the various NGS methods, we chose the 454 GS-FLX system primarily due to its ability to produce long sequence reads [24], since at the time of this study the cod genome assembly had not been published. The 69,440,362 bp of transcriptome sequences obtained in the present study represent a significant contribution to the existing transcriptomic resources for fast skeletal muscle in Atlantic cod. In the clustering step of cleaned sequences prior to RNA-Seq analysis, we obtained several contigs that contained splice variants with different mRNA lengths. This actually highlights the advantage of 3’ cDNA sequencing for distinguishing splice variants and accurately relating sequence read with the copy number of transcribed mRNAs [16]. For gene expression analysis by NGS, Torres et al. [25] demonstrated quantification accuracy in massively parallel sequencing of fragmented 3’ cDNA and that expression data were comparable to microarray experiments in *Drosophila melanogaster*. In addition, Sarropoulou et al. [16] used the same approach to characterise changes in transcriptome profiles in European sea bass (*Dicentrarchus labrax*).

The UniGene cDNA data was selected as the most appropriate reference for our transcriptomic data, since it resulted in a higher annotation rate than Ensembl (3,059 UniGenes versus 1,499 unique and annotated fragments in Ensembl). Nevertheless, 48.5% of contigs could not be annotated and some of them may represent novel transcripts. It is also likely that the current UniGene build (#12, July 2012) does not contain a significant number of transcripts. In addition, the reads obtained in this study always start from 3’ end and contain UTR sequences. Relatively long 3’UTR regions pose a limitation in BLAST searches against reference data from other species due to a generally low conservation of 3’ UTRs.

The category “binding protein” was the most abundant GO term for molecular function at level 2, as often seen other animal tissues [16, 26]. A considerable decrease in abundance of the term “catalytic activity” was found in March for both sexes, suggesting that this function may be influenced by gonadal maturation. Most highly expressed genes in fast skeletal muscle in Atlantic cod were myofibrillar transcripts, as reported in gilthead seabream [13]. Subsequent heat map analysis based on RPKM values of the top 50 highly expressed genes identified a substantial decrease for many myofibrillar transcripts in March, coinciding with the peak of the spawning season in Atlantic cod. This substantial decrease in March was seen both in males and females, thus suggesting a link between gonadal maturation and downregulation of myofibrillar gene expression during reproductive season.

A striking finding from this study is that there were nine *myh* and two *myl* UniGenes among the 153 DEGs throughout the reproductive cycle. The sarcomere is composed of three filament systems: thick filament, thin filament and titin filament. Myosin, the primary component of thick filaments, is the most abundant protein in skeletal muscle and involved in muscle contraction of animals [27]. Remarkably, seven *myh* and one *myl* genes were substantially downregulated in females in March and their downregulation was more prominent compared to males. This could account at least partly for the substantial reduction in specific growth rate observed in females during the spawning period, namely between February and March. Degradation of Myh proteins in Atlantic cod muscle also leads to textural changes like muscle softening and gaping of fillets during post-mortem storage [28]. Therefore, it is most likely that the substantial downregulation for several *myh* UniGenes contributes to muscle wasting during the spawning season. Phylogenetic analysis revealed that the *myh* sequences obtained from our libraries were all *myh13* isoforms belonging to fast muscle type *myh*. The teleost orthologue of *myh13* gene has only been recently identified *in silico* and it has several paralogues in all fish species examined (three to 10 isoforms in Ensembl, S6 Table). Mammalian *Myh13* was identified as an extraocular myosin isoform which also expressed in the superfast laryngeal muscles [29] and was mapped to the cluster of six fast and developmental myosin heavy chain genes on chromosome 17 [30]. To date, there are no published reports of *myh13* in fish species. It would be particularly relevant to characterise teleost *myh13* and its regulation in fast skeletal muscle by environmental factors. In particular, photoperiod is known to affect expression of key myogenic genes in fast skeletal muscle, as previously reported in our sister paper [21]. In common carp, *Cyprinus carpio*, expression of three other *myh* isoforms (10°C-, intermediate- and 30°C-types) is dependent on acclimation temperature [31]. It is unlikely that changes in transcript levels of *myh* isoforms identified as DEG in the present study were caused by water temperature, since it varied gradually and was kept within a narrow range (4.9 to 10.4°C, Fig 1). Thirteen *ttn* UniGenes were the most abundant transcripts among all 153 DEGs, and they were mainly downregulated in females in March. *Ttn* is one of the most abundant striated-muscle-specific protein that spans half a sarcomere from the Z-disc to M-line [32]. It is the largest molecule (3 to 4 MDa) presently known and its function according to muscle type is maintained by the existence of various isoforms generated by alternative splicing [33]. Knockdown of *ttn* in zebrafish produced morphants with a disrupted sarcomere structure and a severely distorted muscle fibre shape [34]. Hence, it seems plausible that the downregulation of *ttn* genes may be associated with muscle wasting during the spawning period in Atlantic cod.

Verification of the 454 pyrosequencing data by qPCR confirmed the significant downregulation of *myh* and *myl2* in the spawning period. Since *myl2* is expressed in newly formed white muscle fibres [35], downregulated *myl2* may result in the inhibition of hypertrophic growth of white muscle. Moreover, both *actc1* and *acta* paralogues were downregulated in males, suggesting that actin function in skeletal muscle cells (e.g., maintenance of the cytoskeleton, cell motility and muscle contraction [36]) could be impaired during the reproductive period. This marked decrease in *actc1* and *acta* transcript levels at the peak of the reproductive season was not observed in females, suggesting that ovarian and testicular maturation may have a negative impact on muscle function/ growth through different molecular mechanisms. In contrast, transcript levels of a novel *sall* paralogue increased during the reproductive period. In mammals, *sall* homologues encode zinc finger transcription repressors and mice heterozygous for a gene trap allele of *Sall4* show limb and heart defects [37]. In addition, *Sall4* also regulates stem cell maintenance and differentiation of the tissue progenitor cells in mouse heart [37]. There are very few reports of *sall* homologues in fish but Wang et al [38] have shown that *sall4* is highly expressed in undifferentiated cultures of medaka embryonic stem cell lines and significantly downregulated upon induced differentiation by formation of embryoid bodies. It is plausible that an increase of transcript levels of this novel *sall* repressor may affect differentiation of muscle progenitor cells in Atlantic cod.

## Conclusions

3’UTR RNA-Seq revealed dynamic changes in muscle transcriptome profiles throughout a reproductive cycle of adult Atlantic cod. In particular, a substantial downregulation of *myh13* and other myofibrillar genes was observed during the spawning period in fast skeletal muscle. These differentially expressed genes that may be affected by gonadal maturation contribute significantly to our limited understanding of the molecular mechanisms underlying muscle wasting and regeneration during the reproductive cycles of batch spawners. Importantly, they could also be applied in the aquaculture industry as useful indicators for monitoring flesh quality and condition of skeletal muscle in Atlantic cod and perhaps other commercial fish species.

## Supporting Information

S1 Fig Validation of reference cDNA data of Atlantic cod for RNA-Seq analysis. a) Comparison of sequence number for each cDNA data resource. b) Comparison of the annotation ratios using all valid leads.
(TIF)

S2 Fig Direct GO count for all annotated fragments to UniGene. a) Distribution of transcripts in molecular functions. b) Distribution of transcripts in cellular component. c) Distribution of transcripts in biological process.
(PDF)

S3 Fig Heat map for the top 50 highly expressed genes annotated using the Atlantic cod Ensembl cDNA data set. (TIF)

S4 Fig Differentially expressed genes (DEGs) in fast skeletal muscle of Atlantic cod between sampling points within sex. X- and y-axis indicate RPKM values for all pairwise comparisons. a) Female between August and March. b) Female between March and May. c) Female between August and May. d) Male between August and March. e) Male between March and May. f) Male between August and May. DEGs were determined by pairwise comparison when their log_2_ fold change was log_2_ Ratio > 2 (upregulated gene) or log_2_ Ratio < -2 (downregulated gene). Green circles represent upregulated DEGs and red circles indicate downregulated DEGs. Grey circles show the UniGenes that did not change significantly.
(PDF)

S5 Fig Correlation analysis between qPCR and 454 pyrosequencing data. Mean transcript levels obtained by qPCR data and RPKM values from RNA-seq analysis are represented for the five DEGs examined. Spearman's linear regression coefficient between qPCR data and RPKM values (n = 30) was 0.75 (p < 0.0001), indicating a strong correlation between them.
(TIFF)

S1 Table Top 50 highly expressed transcripts in Ensembl based on RKPM and NCBI blastx matches. (DOC)

S2 Table Overview of gene ontology (GO) functional annotation analysis for each library. (DOC)

S3 Table The details of the combined graph of gene ontology (GO) functions for each library. (DOC)

S4 Table Differentially expressed genes in fast skeletal muscle of Atlantic cod amongst libraries selected by the changes of expression level (RPKM). The numeric data colored with blue and red show log_2_ fold change between adjacent sampling points of significantly upregulated (log_2_ Ratio > 2) and downregulated genes log_2_ Ratio < -2), respectively (P < 0.001).
(DOC)

S5 Table In silico survey of myosin heavy chain (*myh*) genes in Atlantic cod identified in Ensembl Genome Browser in release 71. (DOC)

S6 Table Teleost myosin heavy chain 13 (*myh13*) genes found in Ensembl Genome Browser in release 71 (April 2013). (DOC)

S7 Table Zebrafish myosin heavy chain (*myh*) genes found in NCBI (June 2013). (DOC)

S8 Table Gene name, contig title/GenBank accession number, contig length, frame, primer sequences (5’ to 3’), amplicon size (bp), PCR efficiency (%) and r^2^ value of the DEGs and endogenous reference genes in Atlantic cod. (DOCX)
