# Supplementary figures and images for: Substantial Downregulation of Myogenic Transcripts in Skeletal Muscle of Atlantic Cod during the Spawning Period
Source: PLoS One. 2016 Feb 4;11(2):e0148374. doi: 10.1371/journal.pone.0148374 (PMC4742245; doi:10.1371/journal.pone.0148374)

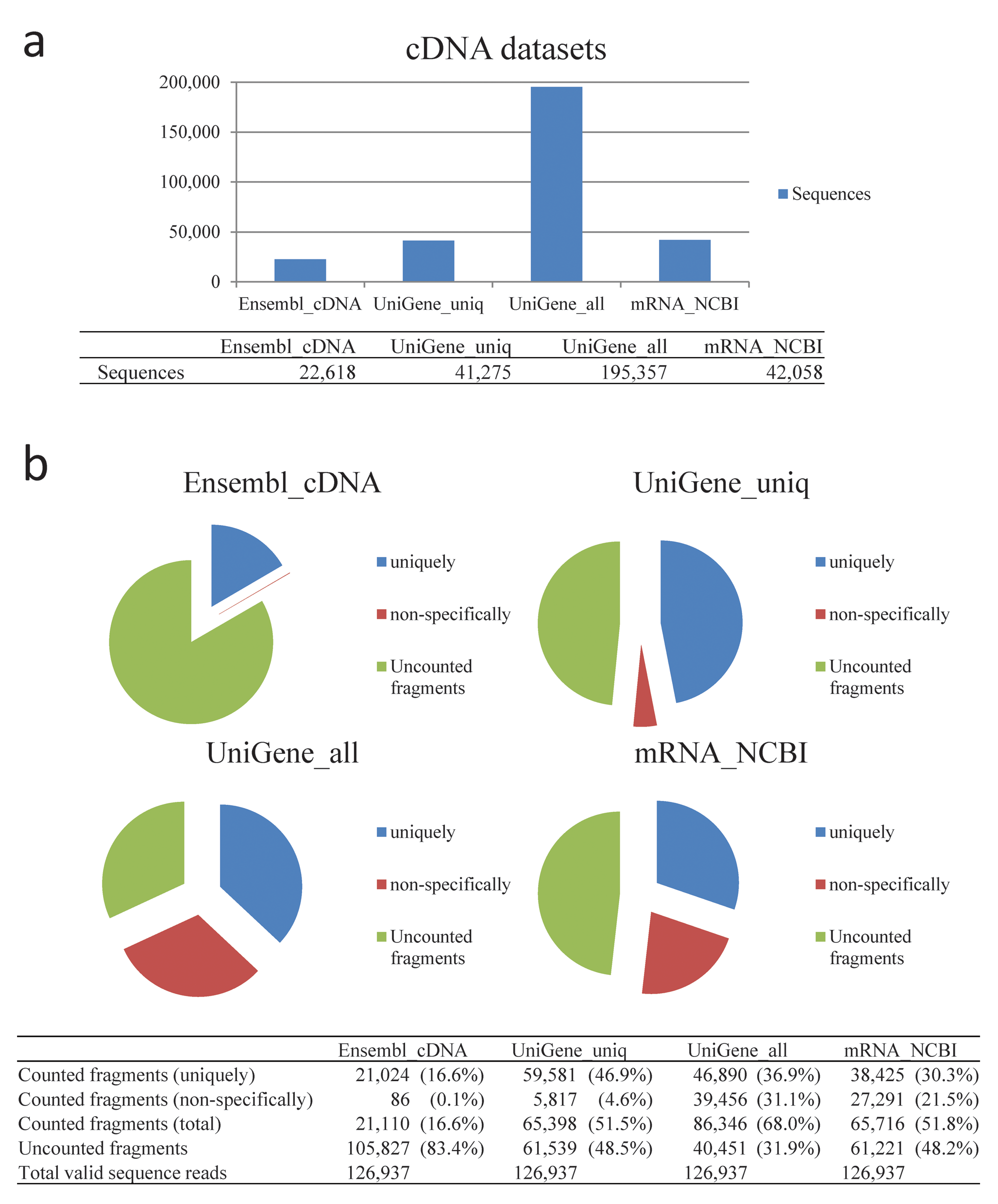

Supplement: S1 Fig — a) Comparison of sequence number for each cDNA data resource. b) Comparison of the annotation ratios using all valid leads. (TIF) [file pone.0148374.s001.tif]

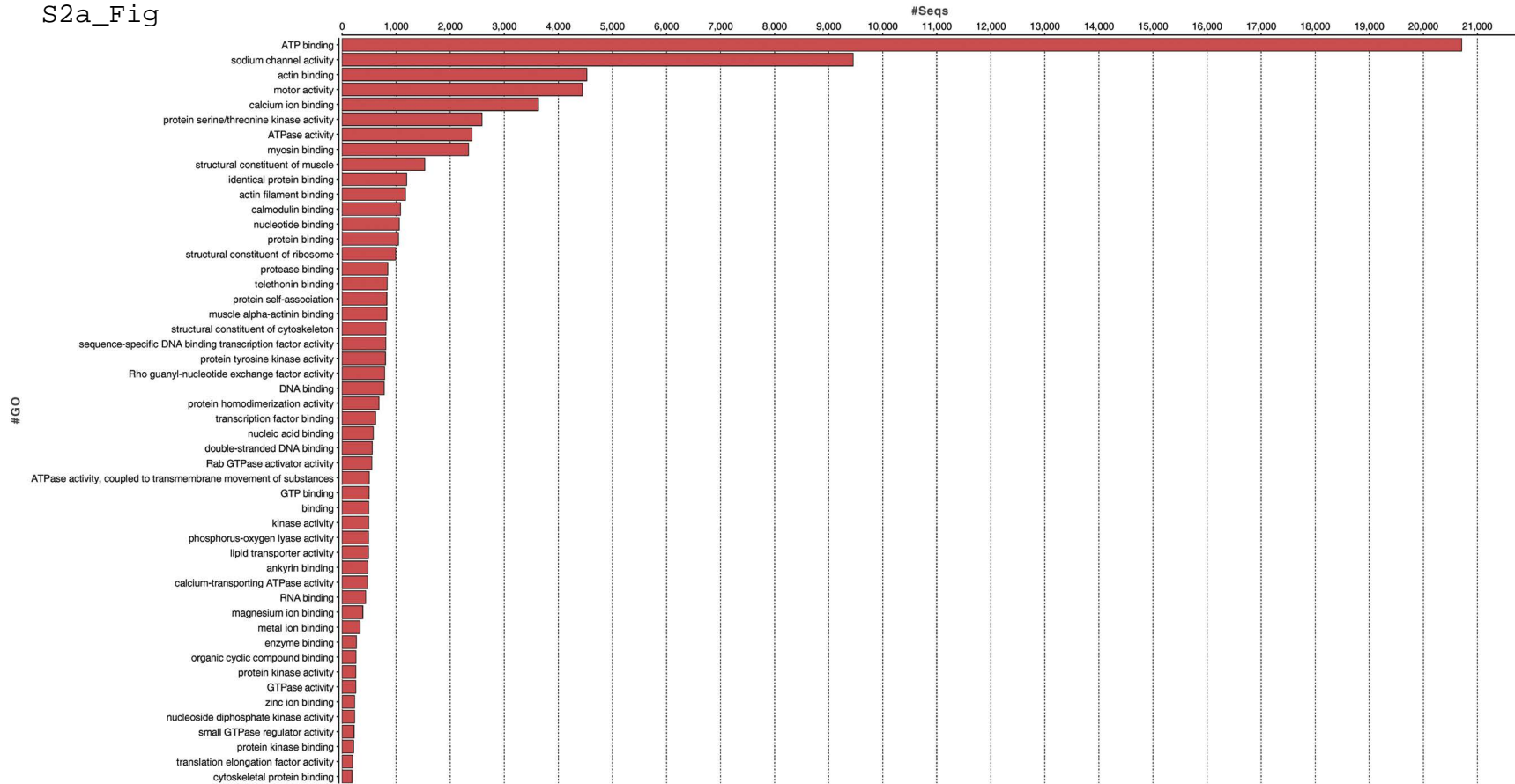

S2b\_Fig

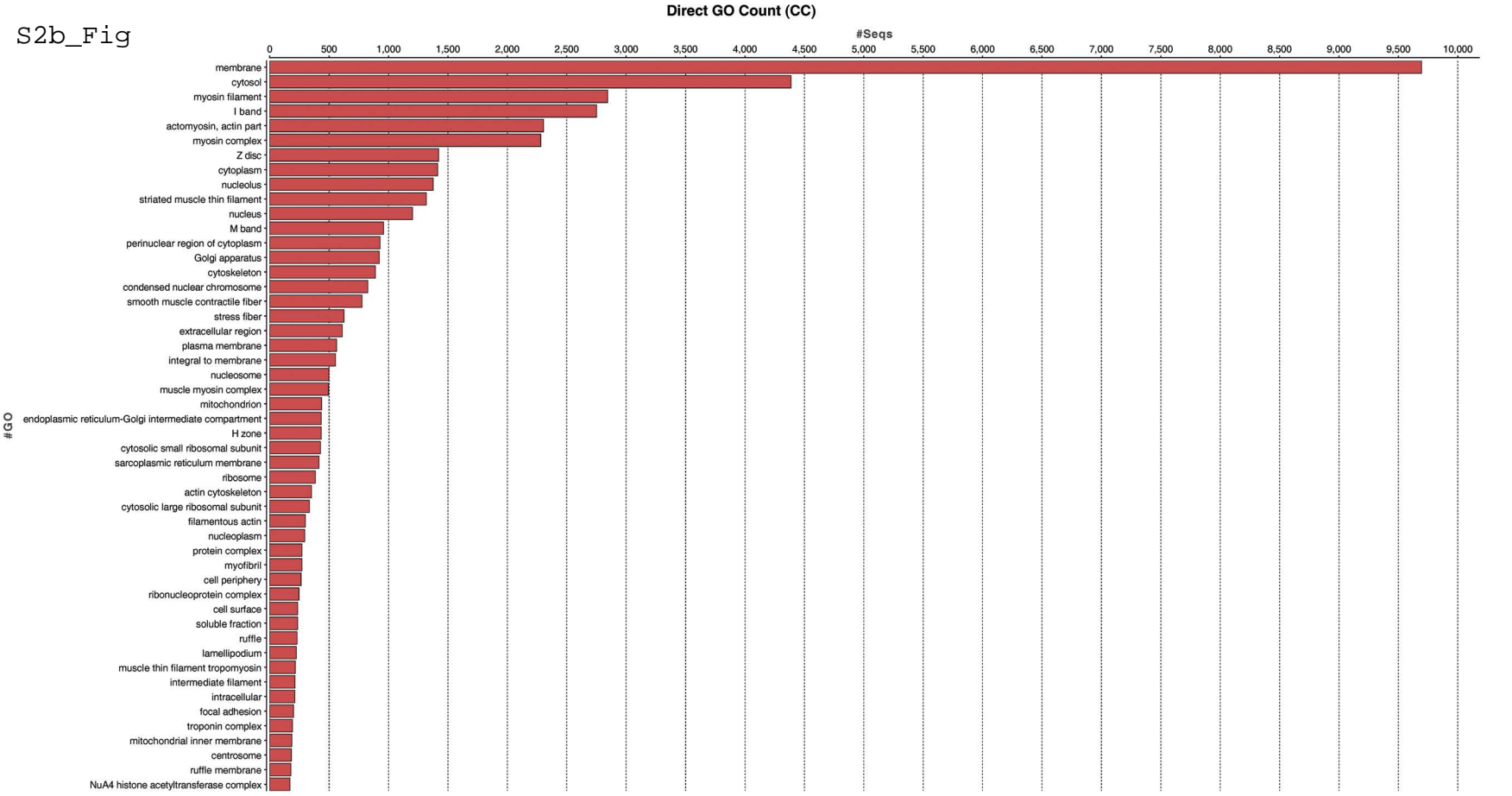

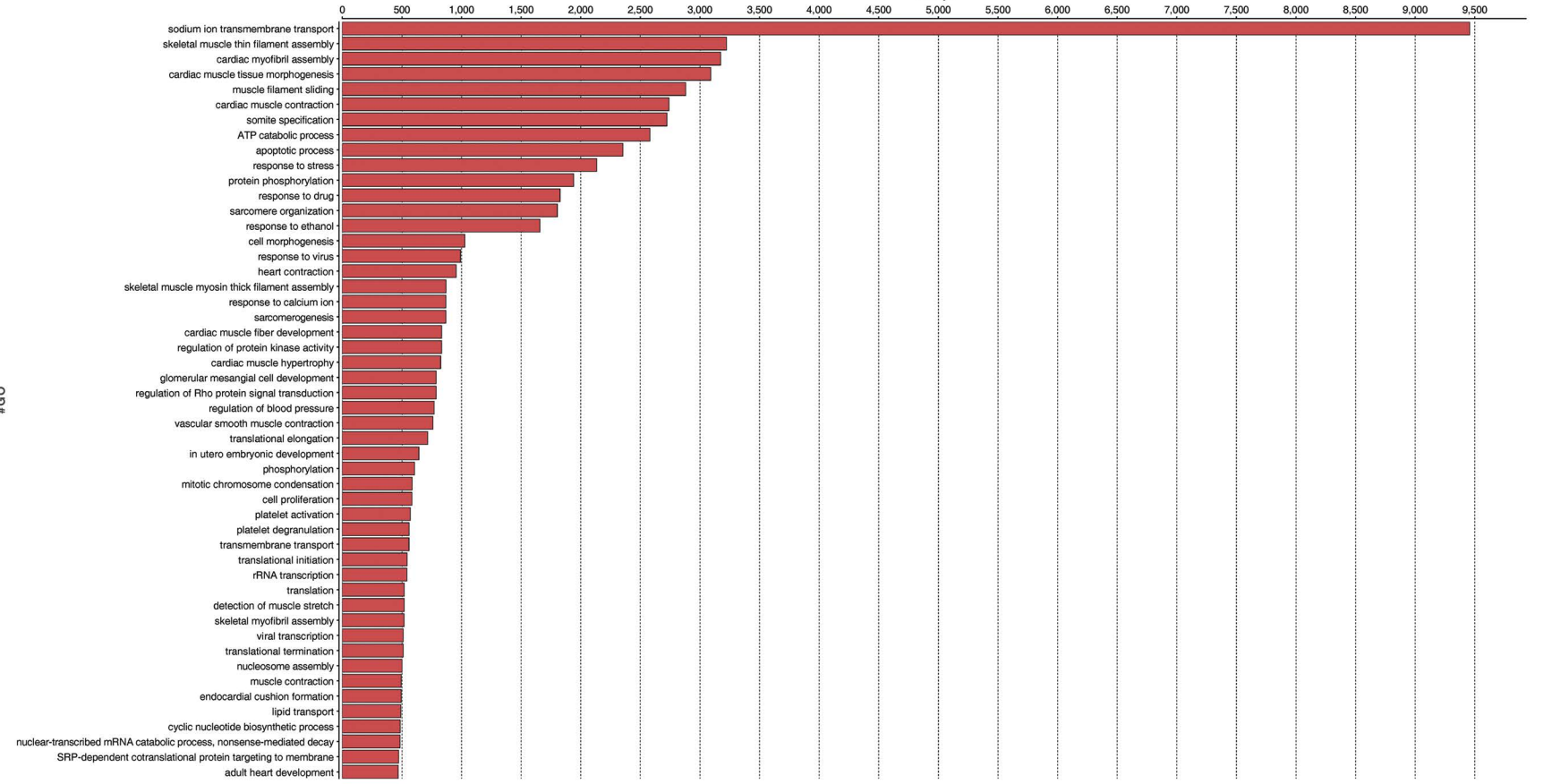

Supplement: S2 Fig — a) Distribution of transcripts in molecular functions. b) Distribution of transcripts in cellular component. c) Distribution of transcripts in biological process. (PDF) [file pone.0148374.s002.pdf]

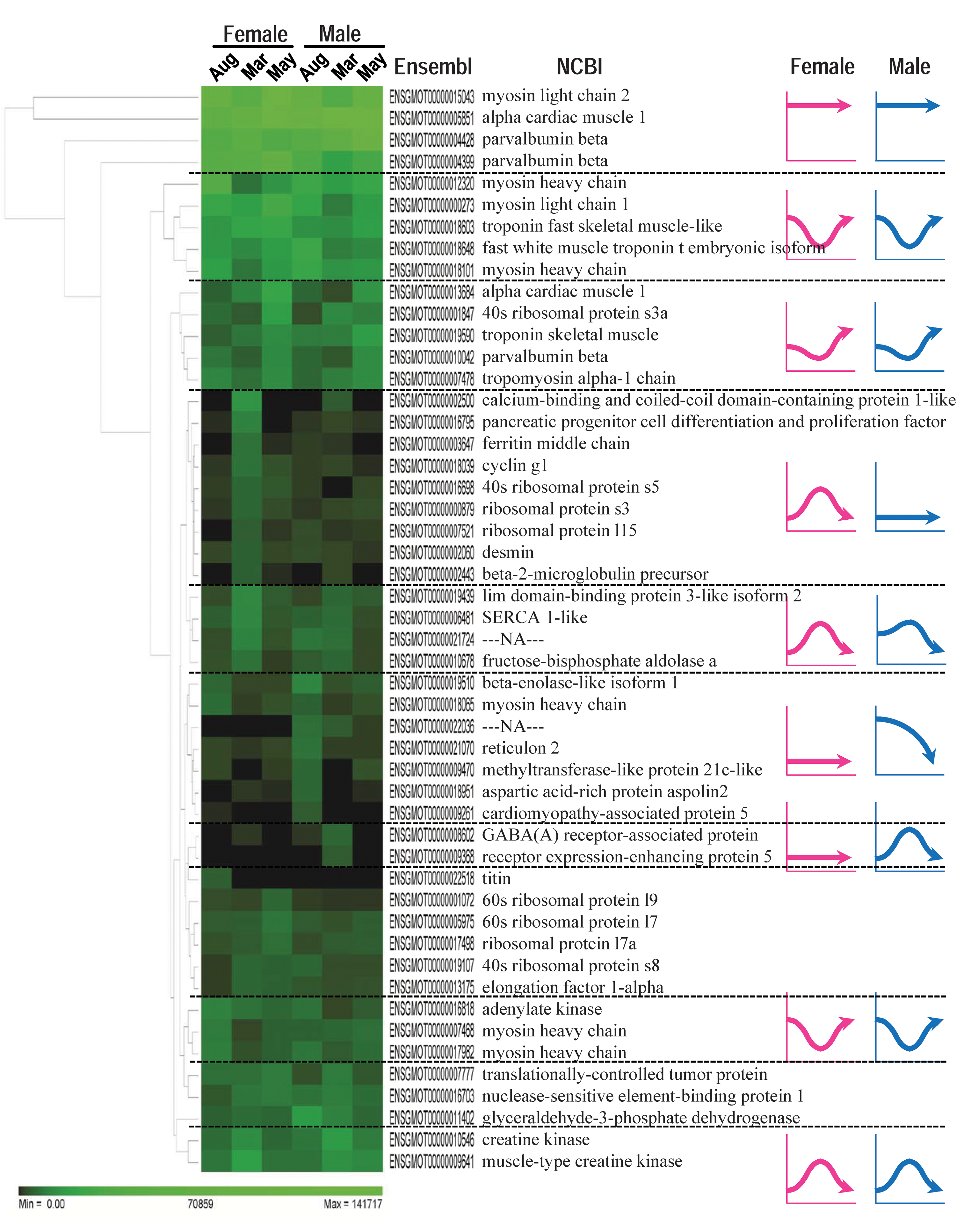

Supplement: S3 Fig — (TIF) [file pone.0148374.s003.tif]

S4a\_Fig

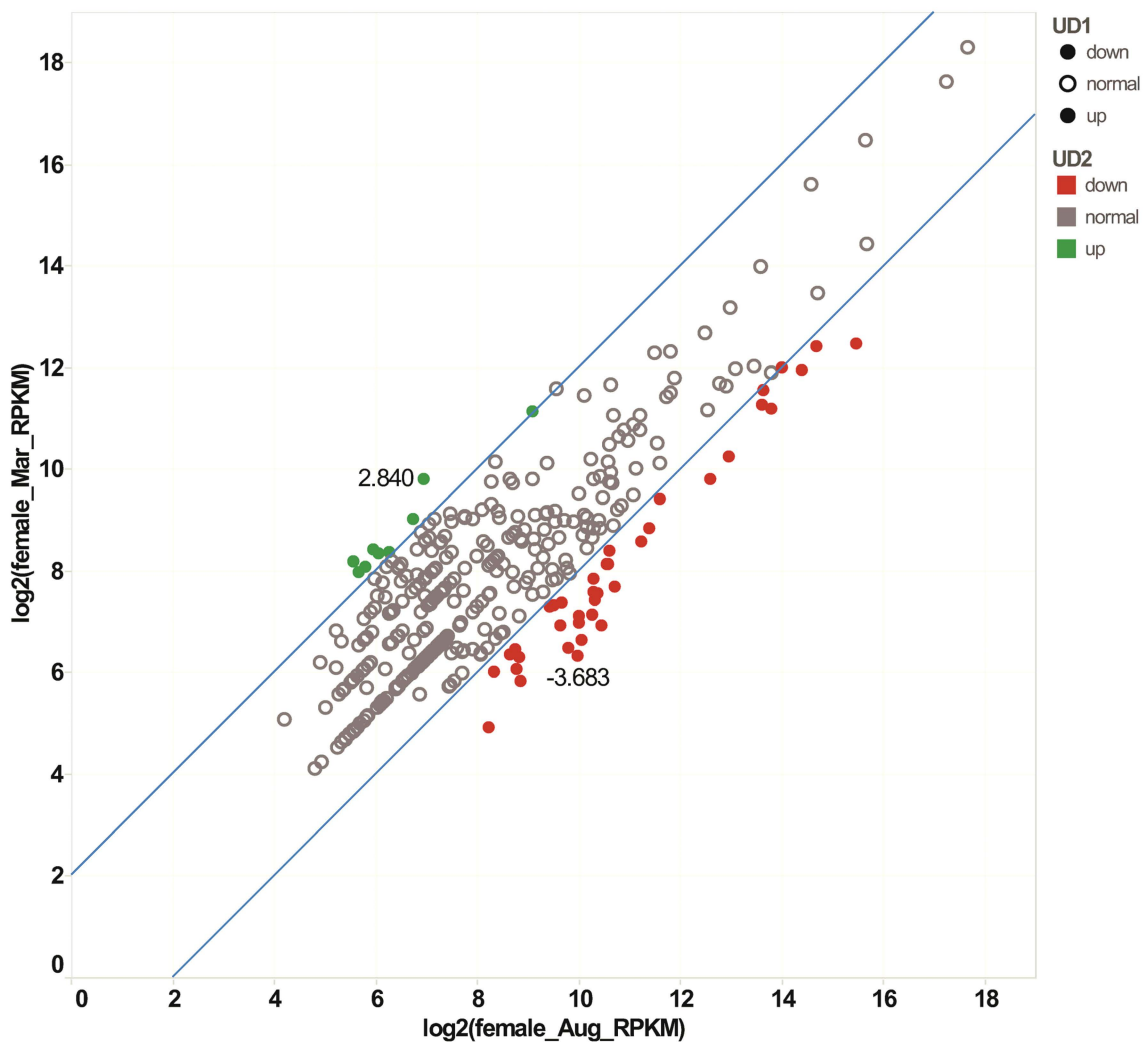

S2b\_Fig

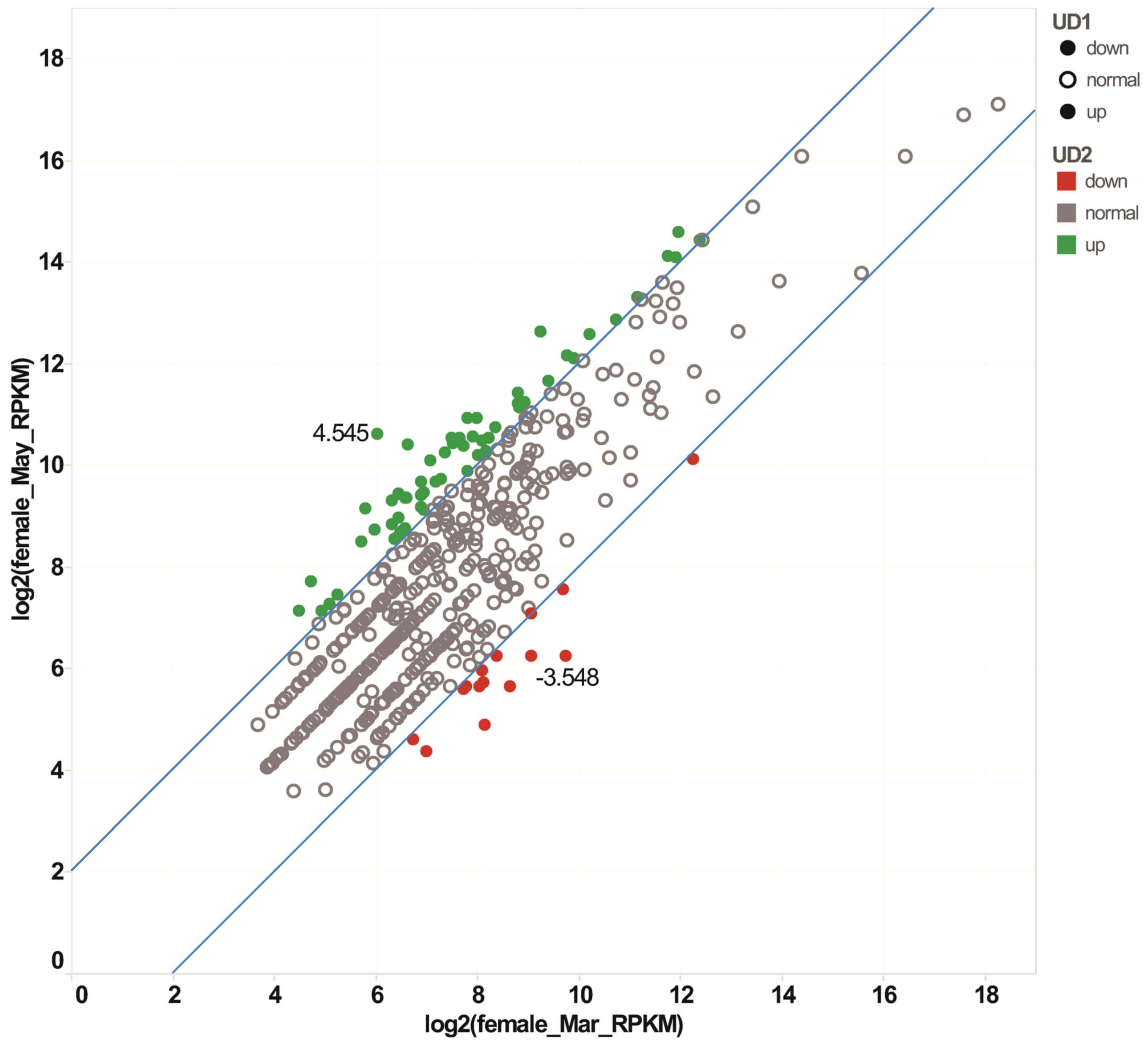

S2c\_Fig

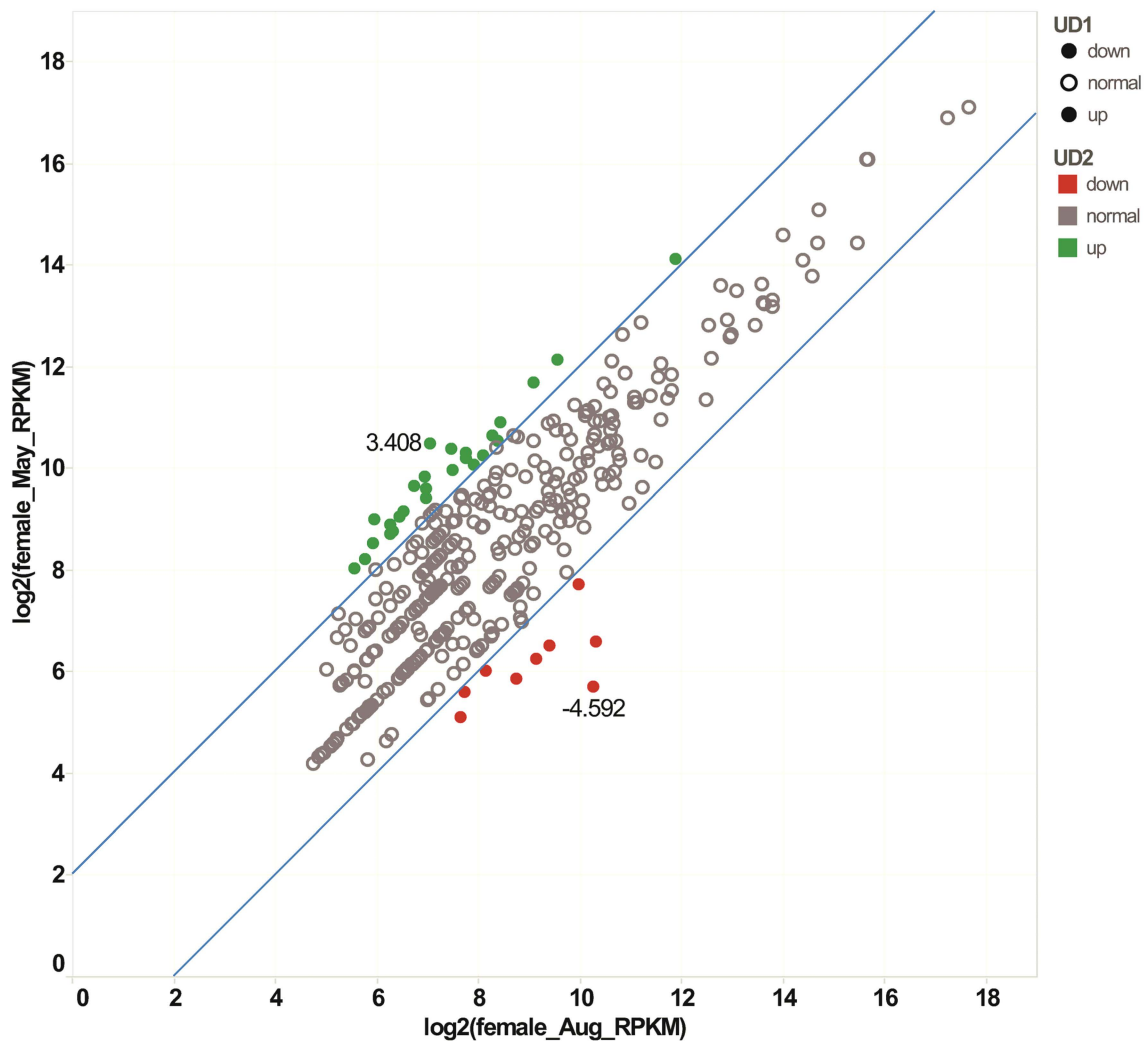

S2d\_Fig

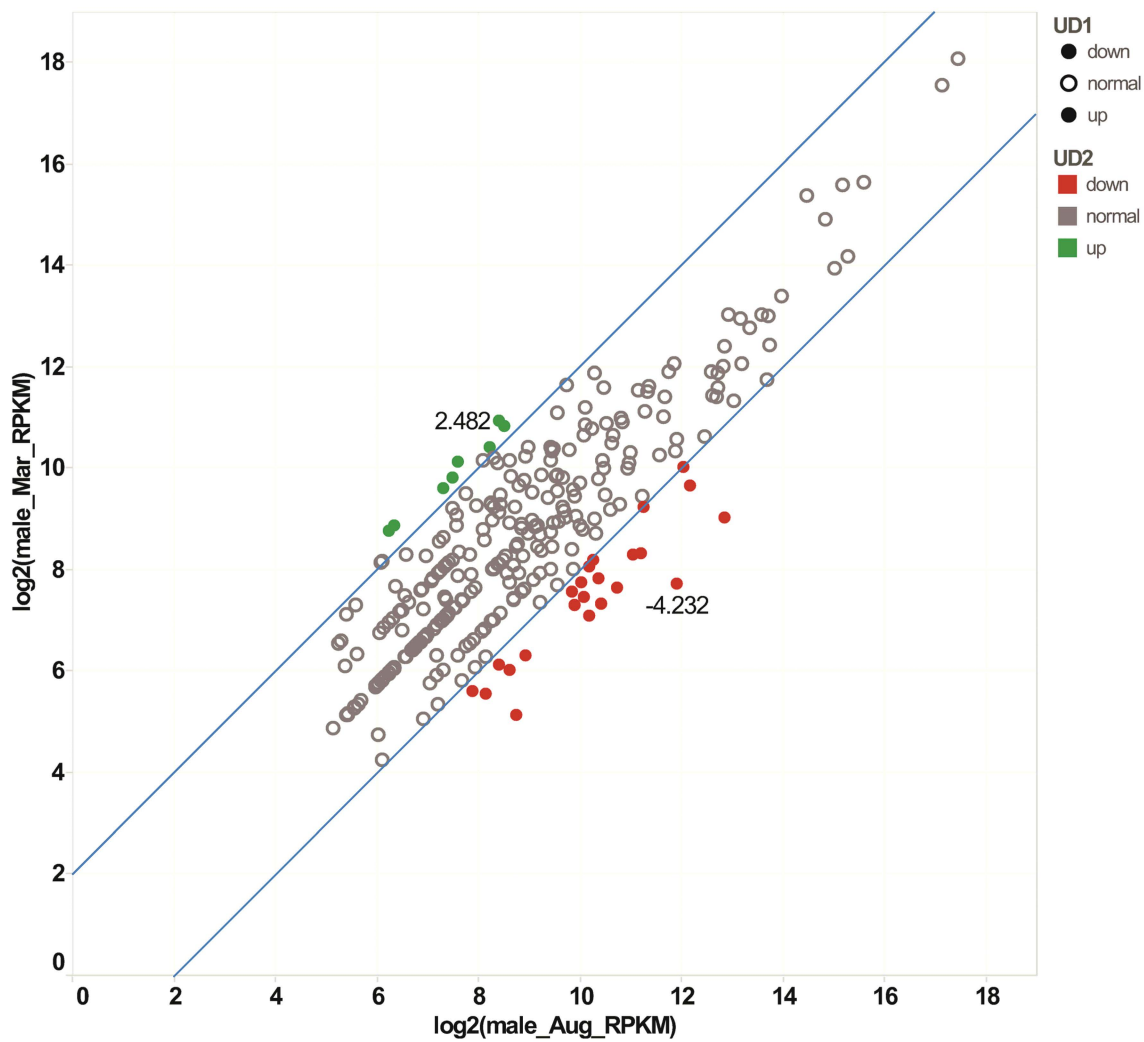

S2e\_Fig

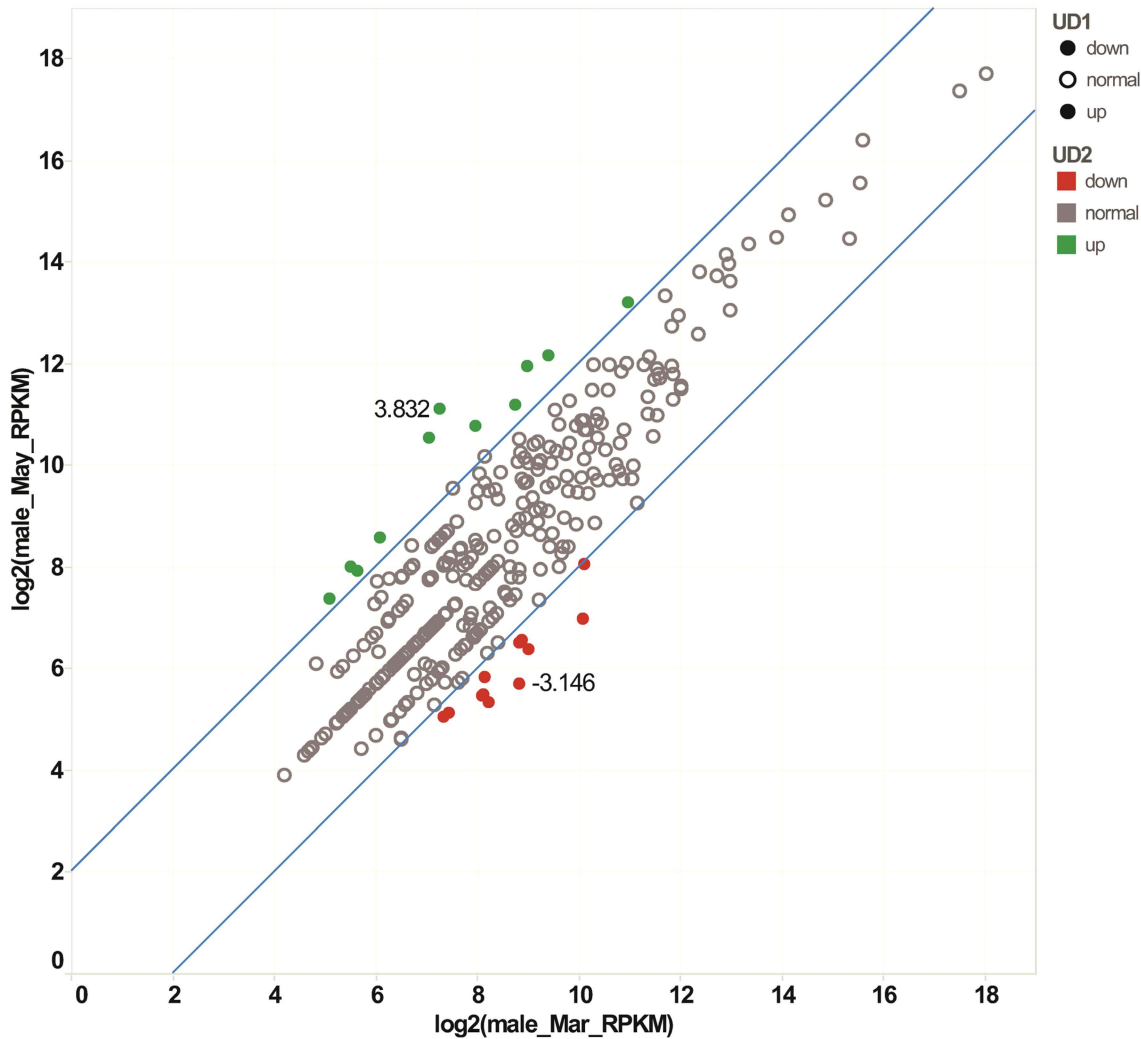

S2f\_Fig

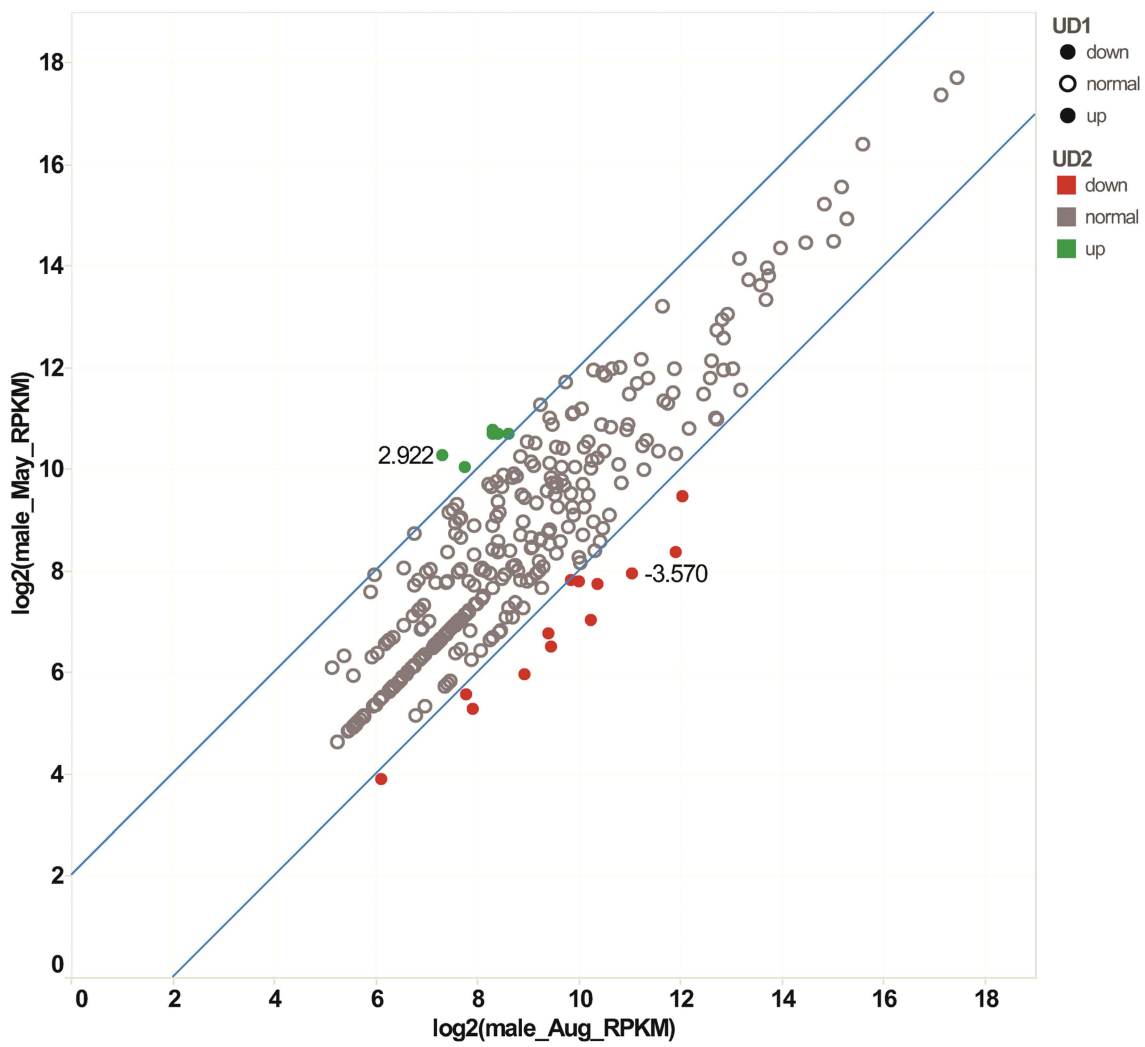

Supplement: S4 Fig — a) Female between August and March. b) Female between March and May. c) Female between August and May. d) Male between August and March. e) Male between March and May. f) Male between August and May. DEGs were determined by pairwise comparison when their log2 fold change was log2 Ratio > 2 (upregulated gene) or log2 Ratio < -2 (downregulated gene). Green circles represent upregulated DEGs and red circles indicate downregulated DEGs. Grey circles show the UniGenes that did not change significantly. (PDF) [file pone.0148374.s004.pdf]

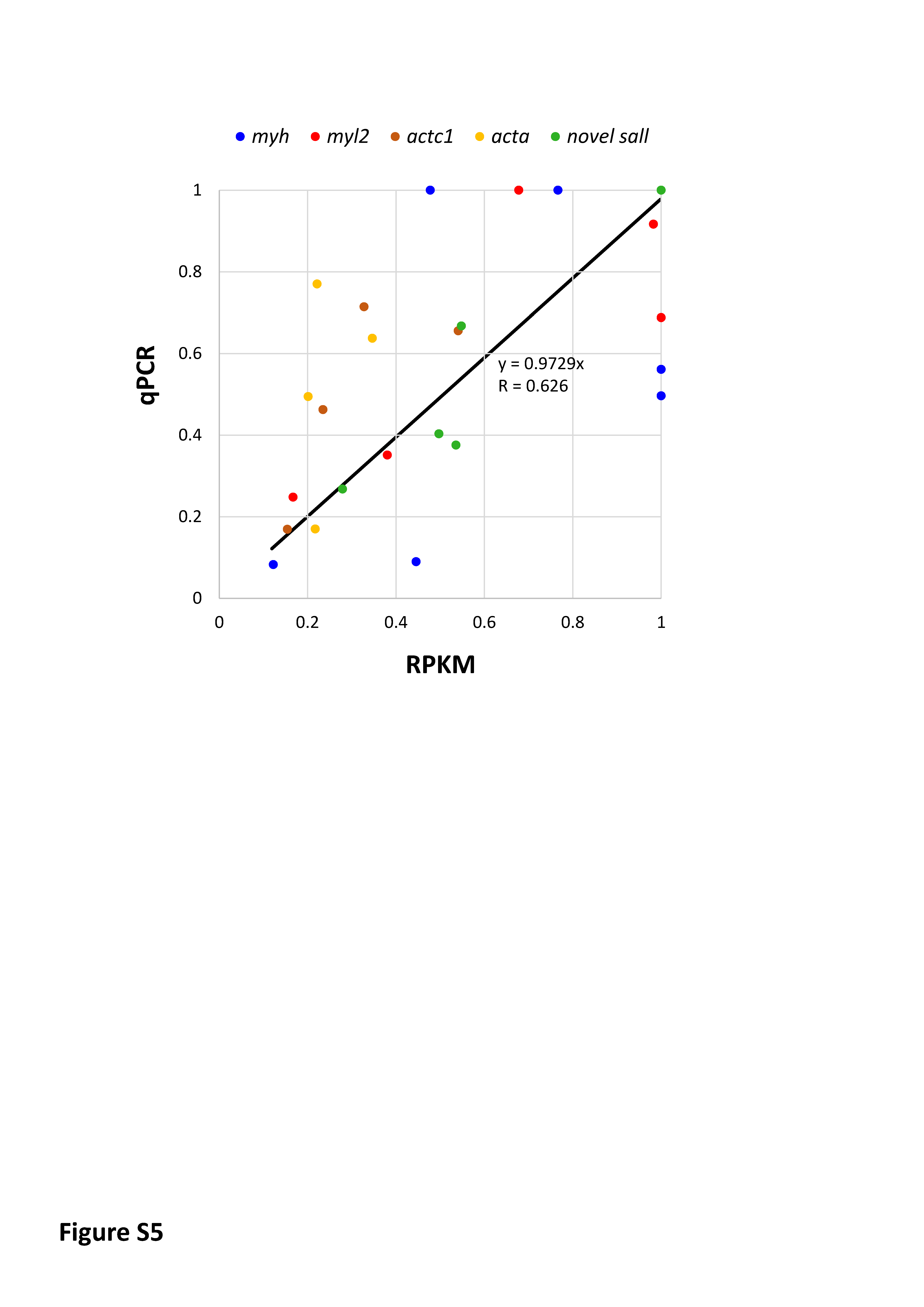

Supplement: S5 Fig — Mean transcript levels obtained by qPCR data and RPKM values from RNA-seq analysis are represented for the five DEGs examined. Spearman's linear regression coefficient between qPCR data and RPKM values (n = 30) was 0.75 (p < 0.0001), indicating a strong correlation between them. (TIFF) [file pone.0148374.s005.tiff]
