# Supplementary material for: Substantial Downregulation of Myogenic Transcripts in Skeletal Muscle of Atlantic Cod during the Spawning Period
Source: PLoS One. 2016 Feb 4;11(2):e0148374. doi: 10.1371/journal.pone.0148374 (PMC4742245; doi:10.1371/journal.pone.0148374)
Supplement: S1 Table — (DOC) [file pone.0148374.s006.doc]

S1 Table. Top 50 highly expressed transcripts in Ensembl according range based on RKPM and NCBI blastx matches.

|  | Seq. Name | Range | p-value Female | p-value Male | Seq. Description | Hit ACC | E-value | Similarity (%) | |  |
| --- | --- | --- | --- | --- | --- | --- | --- | --- | --- | --- |
| 1 | ENSGMOT00000015043 | 96742 | 0 | 0 | myosin light chain 2 | BAB18578 | 1.49E-116 | | 98 | |
| 2 | ENSGMOT00000005851 | 82031 | 0 | 0 | alpha cardiac muscle 1 | AAM21702 | 0 | | 100 | |
| 3 | ENSGMOT00000004428 | 65334 | 0 | 0 | parvalbumin beta | Q90YK9 | 7.02E-61 | | 93 | |
| 4 | ENSGMOT00000004399 | 46708 | 0 | 0 | parvalbumin beta | AAK63086 | 5.70E-51 | | 100 | |
| 5 | ENSGMOT00000012320 | 29931 | 0 | 0 | myosin heavy chain | BAA19070 | 0 | | 98 | |
| 6 | ENSGMOT00000000273 | 21145 | 0 | 0 | myosin light chain 1 | BAA95143 | 2.42E-111 | | 100 | |
| 7 | ENSGMOT00000013684 | 20104 | 0 | 0 | alpha cardiac muscle 1 | NP_001098276 | 0 | | 100 | |
| 8 | ENSGMOT00000001847 | 16645 | 0 | 0 | 40s ribosomal protein s3a | A2Q0R8 | 0 | | 98 | |
| 9 | ENSGMOT00000002500 | 15666 | 0 | 0 | calcium-binding and coiled-coil domain-containing protein 1-like | XP_004069299 | 1.28E-47 | | 80 | |
| 10 | ENSGMOT00000018648 | 15638 | 4.9E-233 | 0 | fast white muscle troponin t embryonic isoform | AAM21701 | 3.62E-66 | | 99 | |
| 11 | ENSGMOT00000018101 | 15341 | 0 | 0 | myosin heavy chain | BAA19070 | 8.62E-150 | | 98 | |
| 12 | ENSGMOT00000019590 | 14094 | 0 | 0 | troponin skeletal muscle | XP_003978216 | 1.18E-89 | | 98 | |
| 13 | ENSGMOT00000011402 | 12875 | 1.5E-79 | 0 | glyceraldehyde-3-phosphate dehydrogenase | AAL05892 | 0 | | 100 | |
| 14 | ENSGMOT00000010546 | 11931 | 0 | 0 | creatine kinase | AAO24738 | 0 | | 94 | |
| 15 | ENSGMOT00000019439 | 11101 | 0 | 0 | lim domain-binding protein 3-like isoform 2 | XP_003441804 | 6.47E-172 | | 89 | |
| 16 | ENSGMOT00000009641 | 10898 | 0 | 0 | muscle-type creatine kinase | ACU11929 | 0 | | 97 | |
| 17 | ENSGMOT00000016795 | 10861 | 0 | 8.7E-214 | pancreatic progenitor cell differentiation and proliferation factor | XP_003963582 | 3.62E-54 | | 85 | |
| 18 | ENSGMOT00000018603 | 10650 | 0 | 1.1E-219 | troponin fast skeletal muscle-like | XP_004069416 | 2.03E-66 | | 93 | |
| 19 | ENSGMOT00000006481 | 9983 | 0 | 0 | sarcoplasmic endoplasmic reticulum calcium atpase 1-like | P70083 | 0 | | 91 | |
| 20 | ENSGMOT00000019510 | 9733 | 0 | 0 | beta-enolase-like isoform 1 | XP_004085855 | 0 | | 96 | |
| 21 | ENSGMOT00000010042 | 9649 | 0 | 0 | parvalbumin beta | Q90YK7 | 8.12E-61 | | 99 | |
| 22 | ENSGMOT00000021724 | 9480 | 0 | 0 | ---NA--- | - | - | | - | |
| 23 | ENSGMOT00000016818 | 8570 | 2.6E-289 | 0 | adenylate kinase | NP_001134467 | 4.29E-125 | | 96 | |
| 24 | ENSGMOT00000007468 | 8296 | 0 | 3.5E-66 | myosin heavy chain | XP_003460579 | 1.47E-170 | | 97 | |
| 25 | ENSGMOT00000007478 | 7958 | 0 | 0 | tropomyosin alpha-1 chain | EMC77437 | 1.44E-163 | | 93 | |
| 26 | ENSGMOT00000003647 | 7886 | 0 | 0 | ferritin middle chain | CBM95498 | 4.96E-86 | | 94 | |
| 27 | ENSGMOT00000018039 | 7714 | 0 | 3.5E-150 | cyclin g1 | AFU80860 | 1.01E-170 | | 88 | |
| 28 | ENSGMOT00000022036 | 7597 | -ND- | 0 | ---NA--- | - | - | | - | |
| 29 | ENSGMOT00000021070 | 7568 | 7.1E-185 | 0 | reticulon 2 | NP_001033316 | 7.80E-80 | | 79 | |
| 30 | ENSGMOT00000010678 | 6848 | 0 | 0 | fructose-bisphosphate aldolase a | XP_003438531 | 0 | | 93 | |
| 31 | ENSGMOT00000017982 | 6845 | 0 | 0 | myosin heavy chain | BAG16354 | 0 | | 79 | |
| 32 | ENSGMOT00000007777 | 6803 | 7.5E-94 | 0 | translationally-controlled tumor protein | ABC59222 | 3.94E-72 | | 80 | |
| 33 | ENSGMOT00000016698 | 6305 | 0 | 0 | 40s ribosomal protein s5 | NP_001153991 | 4.46E-148 | | 100 | |
| 34 | ENSGMOT00000018951 | 6297 | 1.1E-146 | 0 | aspartic acid-rich protein aspolin2 | BAC87890 | 6.15E-12 | | 100 | |
| 35 | ENSGMOT00000016703 | 6196 | 0 | 1.5E-121 | nuclease-sensitive element-binding protein 1 | NP_001098143 | 6.15E-70 | | 93 | |
| 36 | ENSGMOT00000009470 | 5752 | 0 | 0 | methyltransferase-like protein 21c-like | XP_003445821 | 5.38E-97 | | 81 | |
| 37 | ENSGMOT00000008602 | 5654 | 0 | 0 | GABA (A) receptor-associated protein | XP_003966959 | 4.63E-81 | | 98 | |
| 38 | ENSGMOT00000001072 | 5650 | 0 | 9.7E-46 | 60s ribosomal protein l9 | AAH90911 | 2.52E-125 | | 95 | |
| 39 | ENSGMOT00000002443 | 5465 | 0 | 0 | beta-2-microglobulin precursor | CAA10761 | 7.62E-80 | | 99 | |
| 40 | ENSGMOT00000018065 | 5375 | 0 | 0 | myosin heavy chain | BAG16351 | 0 | | 89 | |
| 41 | ENSGMOT00000005975 | 5319 | 0 | 1.1E-72 | 60s ribosomal protein l7 | BAF98652 | 9.10E-149 | | 94 | |
| 42 | ENSGMOT00000000879 | 4916 | 0 | 4.3E-145 | ribosomal protein s3 | XP_003968267 | 2.04E-161 | | 99 | |
| 43 | ENSGMOT00000022518 | 4913 | 0 | -ND- | titin | XP_003458433 | 3.50E-81 | | 94 | |
| 44 | ENSGMOT00000019107 | 4800 | 0 | 0 | 40s ribosomal protein s8 | BAF45896 | 5.38E-101 | | 96 | |
| 45 | ENSGMOT00000013175 | 4710 | 0 | 1.39294E-69 | elongation factor 1-alpha | NP_571338 | 0 | | 97 | |
| 46 | ENSGMOT00000017498 | 4638 | 0 | 6.6E-143 | ribosomal protein l7a | BAF98653 | 4.80E-170 | | 97 | |
| 47 | ENSGMOT00000007521 | 4585 | 0 | 1.0E-247 | ribosomal protein l15 | NP_001003447 | 1.21E-125 | | 99 | |
| 48 | ENSGMOT00000002060 | 4531 | 0 | 2.2E-195 | desmin | XP_003445343 | 0 | | 93 | |
| 49 | ENSGMOT00000009261 | 4395 | 2.6E-116 | 0 | cardiomyopathy-associated protein 5 | XP_003458626 | 1.91E-62 | | 70 | |
| 50 | ENSGMOT00000009368 | 4389 | -ND- | 0 | receptor expression-enhancing protein 5 | XP_003442950 | 8.27E-78 | | 85 | |

Range value was calculated by transcriptomic analysis with CLC. P-value was Sequence description, hit NCBI accession number, e-value, and similarity were obtained by blastx against the NCBI nr database.
