# Supplementary material for: Substantial Downregulation of Myogenic Transcripts in Skeletal Muscle of Atlantic Cod during the Spawning Period
Source: PLoS One. 2016 Feb 4;11(2):e0148374. doi: 10.1371/journal.pone.0148374 (PMC4742245; doi:10.1371/journal.pone.0148374)
Supplement: S2 Table — (DOC) [file pone.0148374.s007.doc]

Table S2. Overview of gene ontology (GO) functional annotation analysis for each library.

|  | Female | | | | | | | |  | Male | | | | | | | | | |  | Total | | | |  |
| --- | --- | --- | --- | --- | --- | --- | --- | --- | --- | --- | --- | --- | --- | --- | --- | --- | --- | --- | --- | --- | --- | --- | --- | --- | --- |
|  | Aug | | Mar | | | May | | |  | Aug | | | | Mar | | | May | | |  |  |
| Annotated to UniGene | 9,039 |  | | 15,146 |  | | 13,625 |  | | |  | 7,191 |  | | 9,008 |  | | 11,389 |  | | |  | 65,398 |  | |
| Blasted | 7,767 | 85.9% | | 13,338 | 88.1% | | 10,928 | 80.2% | | |  | 5,995 | 83.4% | | 7,503 | 83.3% | | 9,382 | 82.4% | | |  | 54,913 | 84.0% | |
| Mapped | 5,095 | 56.4% | | 7,262 | 47.9% | | 7,382 | 54.2% | | |  | 4,159 | 57.8% | | 4,845 | 53.8% | | 6,283 | 55.2% | | |  | 35,026 | 53.6% | |
| Annotated | 4,834 | 53.5% | | 6,792 | 44.8% | | 6,856 | 50.3% | | |  | 4,007 | 55.7% | | 4,600 | 51.1% | | 5,828 | 51.2% | | |  | 32,917 | 50.3% | |
