# Supplementary material for: Substantial Downregulation of Myogenic Transcripts in Skeletal Muscle of Atlantic Cod during the Spawning Period
Source: PLoS One. 2016 Feb 4;11(2):e0148374. doi: 10.1371/journal.pone.0148374 (PMC4742245; doi:10.1371/journal.pone.0148374)
Supplement: S3 Table — (DOC) [file pone.0148374.s008.doc]

S3 Table. The details of the combined graph of gene ontology (GO) functions for each library.

| **Molecular function** | Female | | | | | |  | Male | | | | | |
| --- | --- | --- | --- | --- | --- | --- | --- | --- | --- | --- | --- | --- | --- |
|  | Aug | | Mar | | May | |  | Aug | | Mar | | May | |
| antioxidant activity | - | 0% | 4 | 0% | 3 | 0% |  | 1 | 0% | 5 | 0% | 2 | 0% |
| binding | 4,331 | 49% | 6,045 | 48% | 5,886 | 50% |  | 3,581 | 49% | 4,126 | 47% | 5,173 | 50% |
| catalytic activity | 2,321 | 26% | 2,200 | 17% | 2,896 | 25% |  | 2,088 | 28% | 1,965 | 23% | 2,442 | 23% |
| channel regulator activity | 9 | 0% | 9 | 0% | 16 | 0% |  | 6 | 0% | 4 | 0% | 13 | 0% |
| chemoattractant activity | - | 0% | - | 0% | 1 | 0% |  | - | 0% | - | 0% | - | 0% |
| electron carrier activity | 8 | 0% | 9 | 0% | 7 | 0% |  | 9 | 0% | 22 | 0% | 19 | 0% |
| enzyme regulator activity | 185 | 2% | 194 | 2% | 294 | 3% |  | 152 | 2% | 217 | 3% | 281 | 3% |
| molecular transducer activity | 5 | 0% | 20 | 0% | 8 | 0% |  | 2 | 0% | 11 | 0% | 4 | 0% |
| nucleic acid binding transcription factor activity | 129 | 2% | 253 | 2% | 122 | 1% |  | 144 | 2% | 96 | 1% | 81 | 1% |
| protein binding transcription factor activity | 19 | 0% | 58 | 1% | 60 | 1% |  | 21 | 0% | 36 | 0% | 30 | 0% |
| receptor activity | 13 | 0% | 13 | 0% | 15 | 0% |  | 5 | 0% | 2 | 0% | 12 | 0% |
| receptor regulator activity | 1 | 0% | 2 | 0% | 3 | 0% |  | - | 0% | 1 | 0% | 1 | 0% |
| structural molecule activity | 364 | 4% | 412 | 3% | 861 | 7% |  | 334 | 5% | 365 | 4% | 579 | 6% |
| translation regulator activity | 6 | 0% | 9 | 0% | 23 | 0% |  | 8 | 0% | 6 | 0% | 6 | 0% |
| transporter activity | 1,423 | 16% | 3,396 | 27% | 1,556 | 13% |  | 1,035 | 14% | 1,873 | 22% | 1,786 | 17% |
| total | 8,814 |  | 12,624 |  | 11,751 |  |  | 7,386 |  | 8,729 |  | 10,429 |  |
|  |  |  |  |  |  |  |  |  |  |  |  |  |  |
| **Cellular component** | Female | | | | | |  | Male | | | | | |
|  | Aug | | Mar | | May | |  | Aug | | Mar | | May | |
| cell | 2,510 | 28% | 2,270 | 22% | 4,066 | 30% |  | 2,217 | 29% | 2,127 | 26% | 3,184 | 29% |
| cell junction | 39 | 0% | 56 | 1% | 80 | 1% |  | 46 | 1% | 31 | 0% | 50 | 1% |
| extracellular matrix | 10 | 0% | 8 | 0% | 10 | 0% |  | 3 | 0% | 6 | 0% | 6 | 0% |
| extracellular region | 124 | 1% | 111 | 1% | 185 | 1% |  | 106 | 1% | 110 | 1% | 149 | 1% |
| macromolecular complex | 2,023 | 23% | 1,502 | 15% | 3,013 | 22% |  | 1,747 | 23% | 1,542 | 19% | 2,404 | 22% |
| membrane | 1,474 | 17% | 3,670 | 36% | 1,675 | 13% |  | 1,129 | 15% | 1,971 | 24% | 1,801 | 16% |
| membrane-enclosed lumen | 256 | 3% | 459 | 5% | 623 | 5% |  | 256 | 3% | 362 | 4% | 441 | 4% |
| organelle | 2,377 | 27% | 2,056 | 20% | 3,763 | 28% |  | 2,071 | 27% | 1,975 | 24% | 2,958 | 27% |
| synapse | 10 | 0% | 30 | 0% | 18 | 0% |  | 19 | 0% | 16 | 0% | 15 | 0% |
| total | 8,823 |  | 10,162 |  | 13,433 |  |  | 7,594 |  | 8,140 |  | 11,008 |  |
|  |  |  |  |  |  |  |  |  |  |  |  |  |  |
| **Biological process** | Female | | | | | |  | Male | | | | | |
|  | Aug | | Mar | | May | |  | Aug | | Mar | | May | |
| biological adhesion | 57 | 0% | 107 | 1% | 104 | 1% |  | 58 | 1% | 66 | 0% | 60 | 0% |
| biological regulation | 736 | 5% | 1,157 | 6% | 1,432 | 6% |  | 721 | 6% | 885 | 6% | 1,044 | 6% |
| cell proliferation | 153 | 1% | 316 | 2% | 176 | 1% |  | 152 | 1% | 144 | 1% | 96 | 1% |
| cellular component organization or biogenesis | 922 | 7% | 1,056 | 5% | 1,922 | 9% |  | 877 | 7% | 1,009 | 7% | 1,438 | 8% |
| cellular process | 3,008 | 22% | 5,772 | 28% | 4,413 | 20% |  | 2,585 | 21% | 3,548 | 24% | 3,719 | 21% |
| death | 466 | 3% | 671 | 3% | 936 | 4% |  | 460 | 4% | 588 | 4% | 666 | 4% |
| developmental process | 1,570 | 12% | 1,274 | 6% | 2,530 | 11% |  | 1,397 | 11% | 1,364 | 9% | 2,128 | 12% |
| growth | 48 | 0% | 51 | 0% | 75 | 0% |  | 49 | 0% | 50 | 0% | 78 | 0% |
| immune system process | 41 | 0% | 193 | 1% | 120 | 1% |  | 62 | 1% | 98 | 1% | 43 | 0% |
| localization | 1,627 | 12% | 3,805 | 18% | 2,082 | 9% |  | 1,239 | 10% | 2,130 | 14% | 2,106 | 12% |
| locomotion | - | 0% | 125 | 1% | 211 | 1% |  | 100 | 1% | 136 | 1% | 175 | 1% |
| metabolic process | 1,511 | 11% | 2,380 | 12% | 2,552 | 11% |  | 1,478 | 12% | 1,674 | 11% | 1,705 | 10% |
| multi-organism process | 242 | 2% | 334 | 2% | 462 | 2% |  | 243 | 2% | 260 | 2% | 315 | 2% |
| multicellular organismal process | 1,535 | 11% | 1,384 | 7% | 2,418 | 11% |  | 1,378 | 11% | 1,397 | 9% | 2,066 | 12% |
| pigmentation | - | 0% | - | 0% | - | 0% |  | - | 0% | - | 0% | - | 0% |
| reproduction | 94 | 1% | 226 | 1% | 284 | 1% |  | 82 | 1% | 123 | 1% | 138 | 1% |
| response to stimulus | 1,229 | 9% | 1,184 | 6% | 1,847 | 8% |  | 1,051 | 9% | 1,130 | 8% | 1,558 | 9% |
| rhythmic process | - | 0% | - | 0% | - | 0% |  | - | 0% | - | 0% | - | 0% |
| signaling | 291 | 2% | 495 | 2% | 495 | 2% |  | 246 | 2% | 386 | 3% | 390 | 2% |
| viral reproduction | 63 | 1% | 198 | 1% | 251 | 1% |  | 66 | 1% | 97 | 1% | 113 | 1% |
| total | 13,593 |  | 20,728 |  | 22,310 |  |  | 12,244 |  | 15,085 |  | 17,838 |  |
