# Supplementary material for: Substantial Downregulation of Myogenic Transcripts in Skeletal Muscle of Atlantic Cod during the Spawning Period
Source: PLoS One. 2016 Feb 4;11(2):e0148374. doi: 10.1371/journal.pone.0148374 (PMC4742245; doi:10.1371/journal.pone.0148374)
Supplement: S4 Table — The numeric data colored with blue and red show log2 fold change between adjacent sampling points of significantly upregulated (log2 Ratio > 2) and downregulated genes log2 Ratio < -2), respectively (P < 0.001). (DOC) [file pone.0148374.s009.doc]

S4 Table. Differentially expressed genes in fast skeletal muscle of Atlantic cod amongst libraries selected by the changes of expression level (RPKM). The numeric data colored with blue and red show log_2_ fold change between adjacent sampling points of significantly upregulated (|log_2_ Ratio| > 2) and downregulated genes (-2 > |log_2_ Ratio|), respectively (P < 0.001).

| Seq. Name | | Seq. Description | Hit ACC | Female | | | | | | Male | | | | | |
| --- | --- | --- | --- | --- | --- | --- | --- | --- | --- | --- | --- | --- | --- | --- | --- |
|  |  |  |  | Aug-Mar | P-value | Mar-May | P-value | May-Aug | P-value | Aug-Mar | P-value | Mar-May | P-value | May-Aug | P-value |
| 1 | gnl|UG|Gmr#S60845382 | 14-3-3 protein epsilon | NP_997770 | -ND- | 1.57E-16 | **2.152766** | 3.37E-30 | -ND- | 4.42E-40 | -ND- | 1.31E-13 | -ND- | 2.04E-17 | 0.336605 | 1.35E-07 |
| 2 | gnl|UG|Gmr#S60863280 | 26s protease regulatory subunit 6b | XP_003451328 | -ND- | 8.87E-64 | **-2.16931** | 5.44E-48 | -ND- | 9.38E-26 | -ND- | -ND- | -ND- | -ND- | -ND- | -ND- |
| 3 | gnl|UG|Gmr#S60863377 | 26s protease regulatory subunit 7 | XP_003978561 | -ND- | 6E-127 | **-3.01714** | 8.8E-109 | -ND- | 1.07E-49 | -ND- | 3.23E-84 | -ND- | 3.23E-84 | -ND- | 9.29E-35 |
| 4 | gnl|UG|Gmr#S60868422 | 26s protease regulatory subunit 8 | ACQ58243 | -ND- | 7.87E-83 | **-2.43218** | 1.9E-65 | -ND- | 7.53E-33 | -ND- | 2.02E-30 | -ND- | 2.02E-30 | -ND- | 4.17E-13 |
| 5 | gnl|UG|Gmr#S60867102 | 26s proteasome non-atpase regulatory subunit 11 | CBN81693 | -ND- | 1.47E-31 | **-2.16932** | 6.13E-24 | -ND- | 3.48E-13 | -ND- | 4.56E-28 | -ND- | 4.56E-28 | -ND- | 3.73E-12 |
| 6 | gnl|UG|Gmr#S60866122 | 26s proteasome non-atpase regulatory subunit 4 | CAG10849 | -ND- | 7.34E-87 | -ND- | 7.34E-87 | -ND- | 8.13E-36 | **2.482308** | 7.09E-92 | **-3.14581** | 1.4E-100 | -0.66351 | 5.5E-40 |
| 7 | gnl|UG|Gmr#S44179546 | 40s ribosomal protein s18 | EDL00865 | -0.74475 | 3.17E-43 | **2.474649** | 3.4E-110 | 1.729899 | 2.03E-91 | -ND- | -ND- | -ND- | -ND- | -ND- | -ND- |
| 8 | gnl|UG|Gmr#S55477883 | 40s ribosomal protein s2 | NP_001187067 | 1.42525 | 1.39E-56 | 0.982752 | 1.39E-74 | **2.408002** | 1.2E-126 | -1.32508 | 3.4E-14 | 1.24667 | 7.21E-13 | -0.07841 | 2.55E-06 |
| 9 | gnl|UG|Gmr_S55460118 | 40s ribosomal protein s3a | A2Q0R8 | -1.57477 | 0 | **3.340302** | 0 | 1.765532 | 0 | 1.844903 | 2.4E-263 | 0.076674 | 2.3E-112 | 1.921578 | 3.6E-287 |
| 10 | gnl|UG|Gmr_S55460115 | 40s ribosomal protein s3a | XP_003449725 | -0.96708 | 2.2E-123 | **2.268146** | 0 | 1.301063 | 9.7E-224 | **2.482327** | 5.6E-221 | -0.23883 | 5.97E-86 | **2.243498** | 7.6E-174 |
| 11 | gnl|UG|Gmr#S60843103 | 40s ribosomal protein s4 | ACO07465 | -1.74471 | 3.37E-30 | **2.474607** | 1.83E-57 | 0.729894 | 5.07E-29 | **-2.32505** | 2.22E-38 | **2.46903** | 3.53E-44 | 0.143976 | 6.38E-18 |
| 12 | gnl|UG|Gmr#S60860882 | 40s ribosomal protein s4 | ACO07465 | -ND- | 4.04E-26 | **2.960248** | 6.37E-56 | -ND- | 1.51E-65 | -0.32502 | 9.28E-05 | -1.33829 | 4.46E-07 | -1.66331 | 7.18E-10 |
| 13 | gnl|UG|Gmr#S60861611 | 40s ribosomal protein s8 | BAF45896 | 0.42525 | 1.82E-25 | 1.070217 | 3.82E-50 | 1.495468 | 1.25E-63 | **-2.32499** | 3E-166 | 0.398561 | 2.48E-65 | -1.92643 | 8.6E-151 |
| 14 | gnl|UG|Gmr#S60849620 | 6- muscle type-like | XP_003963241 | -ND- | 1.8E-210 | -ND- | 1.19E-81 | **-2.91395** | 5.3E-178 | -0.32502 | 0.005153 | 0.246625 | 0.018949 | -0.07839 | 0.078966 |
| 15 | gnl|UG|Gmr#S41369811 | 60s ribosomal protein l10 | AAV31599 | -0.74471 | 1.37E-88 | **2.853133** | 5.7E-228 | **2.108421** | 5E-199 | -0.32497 | 0.005182 | -0.33836 | 0.008663 | -0.66333 | 0.00013 |
| 16 | gnl|UG|Gmr_S55475873 | 60s ribosomal protein l11 | ACI12869 | -ND- | 7.2E-128 | **2.027131** | 4.6E-234 | -ND- | 0 | -0.32498 | 0.001544 | 0.661661 | 3.03E-06 | 0.336678 | 0.00055 |
| 17 | gnl|UG|Gmr#S60842461 | 60s ribosomal protein l13 | ACQ58287 | 1.255332 | 1.15E-17 | 1.152696 | 8.03E-27 | **2.408028** | 1.14E-40 | -1.90996 | 3.57E-24 | 0.661587 | 2.96E-10 | -1.24837 | 1.08E-18 |
| 18 | gnl|UG|Gmr#S60844735 | 60s ribosomal protein l13a | CAK04157 | -0.42273 | 6.62E-22 | 1.4157 | 3.33E-54 | 0.992967 | 7.18E-42 | **-2.32503** | 1.3E-150 | 0.246613 | 5.62E-60 | **-2.07842** | 2.3E-142 |
| 19 | gnl|UG|Gmr#S60862217 | 60s ribosomal protein l18a | ACN10349 | 0.255163 | 7.88E-29 | **2.152762** | 2.39E-67 | **2.407925** | 4.66E-71 | -1.32493 | 3.58E-13 | 0.661613 | 1.57E-06 | -0.66332 | 2.57E-08 |
| 20 | gnl|UG|Gmr#S55478924 | 60s ribosomal protein l19 | BAF98668 | 0.840282 | 2.24E-49 | 1.737656 | 3.9E-103 | **2.577938** | 3.4E-126 | -0.32501 | 3.37E-11 | -1.33838 | 1.12E-17 | -1.66338 | 1.46E-25 |
| 21 | gnl|UG|Gmr#S60860901 | 60s ribosomal protein l4-like isoform 2 | ACO09148 | 1.577274 | 1.98E-33 | 1.000688 | 4.92E-43 | **2.577962** | 2.16E-73 | 1.675013 | 1.03E-65 | 0.568536 | 1.29E-52 | **2.243549** | 6.3E-115 |
| 22 | gnl|UG|Gmr_S55469211 | 60s ribosomal protein l7 | ACH70988 | 0.577249 | 1E-118 | 1.875133 | 4.9E-267 | **2.452382** | 1.5E-306 | -0.90997 | 1.38E-33 | 0.983554 | 6.85E-38 | 0.073587 | 1.57E-15 |
| 23 | gnl|UG|Gmr_S44180627 | 60s ribosomal protein l9 | NP_001003861 | -1.74463 | 3.2E-114 | **3.737576** | 2.5E-294 | 1.992947 | 6.5E-230 | -1.32497 | 2.32E-26 | 0.661604 | 2.29E-12 | -0.66336 | 4.07E-16 |
| 24 | gnl|UG|Gmr_S60862237 | adenylate kinase | NP_001134467 | -1.8378 | 9.4E-155 | 1.053127 | 6.9E-71 | -0.78467 | 1.19E-87 | **-2.17301** | 1.2E-130 | 1.42717 | 1.02E-64 | -0.74584 | 6.29E-68 |
| 25 | gnl|UG|Gmr_S60864027 | alpha cardiac muscle 1 | NP_001098276 | -0.48166 | 0 | **2.091277** | 0 | 1.609614 | 0 | -1.81044 | 1.2E-293 | **2.697256** | 0 | 0.886819 | 0 |
| 26 | gnl|UG|Gmr_S44200679 | alpha skeletal muscle | AAA29846 | -0.13702 | 0 | **2.312553** | 0 | **2.175531** | 0 | -0.67294 | 0 | **2.202204** | 0 | 1.529267 | 0 |
| 27 | gnl|UG|Gmr#S60844033 | alpha-actinin-1 isoform 2 | NP_001161758 | -0.32975 | 1.62E-14 | 1.853192 | 2.98E-35 | 1.523446 | 2.38E-31 | **-3.64685** | 5.86E-85 | **2.246497** | 4.92E-36 | -1.40035 | 3.31E-55 |
| 28 | gnl|UG|Gmr_S55478779 | amp deaminase 1 | NP_957187 | 1.992257 | 0 | 0.55867 | 3.1E-239 | **2.550927** | 0 | 1.037559 | 7.6E-158 | -1.92334 | 3.4E-237 | -0.88578 | 2.96E-97 |
| 29 | gnl|UG|Gmr#S55478778 | amp deaminase 1 | AAW52504 | 1.757795 | 4.31E-90 | -0.23435 | 6.97E-36 | 1.523447 | 1.65E-68 | 1.996923 | 1.2E-155 | **-2.07532** | 6.8E-159 | -0.0784 | 4.26E-64 |
| 30 | gnl|UG|Gmr#S60865349 | atpase 6 | NP_008618 | **-2.9146** | 5.9E-135 | **2.960005** | 7.5E-141 | 0.045404 | 2E-56 | -0.74005 | 6.63E-16 | 0.884013 | 2.1E-20 | 0.143963 | 9.09E-09 |
| 31 | gnl|UG|Gmr#S41438663 | beta-2-microglobulin precursor | CAA10761 | -ND- | 9.6E-148 | **-2.01723** | 7.9E-107 | -ND- | 5.63E-59 | 1.259968 | 2.81E-52 | -1.92333 | 2.97E-68 | -0.66336 | 2.21E-27 |
| 32 | gnl|UG|Gmr_S60869000 | beta-enolase | XP_004085855 | **-2.6989** | 0 | 1.059565 | 1.9E-156 | -1.63933 | 0 | **-2.55768** | 0 | 1.147071 | 4E-274 | -1.41061 | 0 |
| 33 | gnl|UG|Gmr_S60857401 | calsequestrin-1 precursor | XP_003457488 | **-2.74472** | 0 | -ND- | 3.4E-146 | -ND- | 0 | **-2.91** | 0 | -ND- | 3.9E-279 | -ND- | 0 |
| 34 | gnl|UG|Gmr_S41425229 | calsequestrin-2 isoform 2 | BAH85793 | **-2.91467** | 8.5E-301 | -0.84732 | 2E-127 | **-3.76199** | 0 | -0.51767 | 1.76E-52 | -1.14572 | 3.98E-61 | -1.66339 | 4.8E-111 |
| 35 | gnl|UG|Gmr#S60849197 | cellular nucleic acid-binding protein | AAW82446 | 1.255389 | 1.22E-13 | 0.737669 | 8.29E-15 | 1.993058 | 7.9E-27 | **-2.64704** | 2.78E-63 | 1.246688 | 5.57E-26 | -1.40035 | 3.77E-45 |
| 36 | gnl|UG|Gmr_S41466882 | chk1 checkpoint-like partial | AEM37715 | -0.44514 | 1.11E-70 | -1.26236 | 2.64E-95 | -1.7075 | 3.5E-160 | **-3.13236** | 1.5E-269 | 1.246564 | 1.9E-104 | -1.88579 | 1.2E-214 |
| 37 | gnl|UG|Gmr#S60867006 | coiled-coil domain-containing protein 43 | XP_003442089 | -ND- | 3.87E-20 | **2.152851** | 4.44E-37 | -ND- | 2.5E-49 | -ND- | 3.37E-14 | 0.661693 | 1.67E-10 | -ND- | 1.56E-22 |
| 38 | gnl|UG|Gmr#S60842702 | cold-inducible rna-binding protein | XP_003914625 | -ND- | 1.94E-39 | 1.612178 | 3.3E-60 | -ND- | 4.99E-93 | -ND- | 3.84E-94 | **-2.92327** | 7.38E-80 | -ND- | 4.33E-37 |
| 39 | gnl|UG|Gmr#S60837547 | comm domain-containing protein 2 | ACQ57998 | -ND- | -ND- | -ND- | -ND- | -ND- | -ND- | **2.482302** | 1.3E-218 | **-3.14568** | 1.3E-239 | -0.66338 | 8.1E-94 |
| 40 | gnl|UG|Gmr#S41386338 | CR1-3 | ADJ80991 | -ND- | 4.16E-31 | -ND- | 4.16E-31 | -ND- | 2.22E-13 | -ND- | 1.5E-203 | -ND- | 5.37E-79 | **-2.66337** | 2E-166 |
| 41 | gnl|UG|Gmr_S60849377 | cyclin g1 | AFU80860 | **2.020828** | 2.2E-237 | 0.54095 | 1E-172 | **2.561778** | 0 | -0.325 | 1.28E-34 | -1.43788 | 1.36E-57 | -1.76289 | 7.42E-84 |
| 42 | gnl|UG|Gmr#S60843164 | cytochrome c oxidase subunit 4 isoform mitochondrial precursor | ACO14190 | -1.15979 | 9.4E-12 | 0.152705 | 2.36E-05 | -1.00709 | 1.2E-10 | **-2.64701** | 2.64E-36 | **2.469076** | 1.06E-30 | -0.17793 | 7.46E-15 |
| 43 | gnl|UG|Gmr#S60871006 | cytochrome c oxidase subunit i | YP_313644 | **-2.55211** | 2.39E-77 | 0.737735 | 1.22E-30 | -1.81437 | 3.4E-65 | 1.259965 | 7.06E-70 | -0.80784 | 1.35E-49 | 0.452123 | 1.23E-28 |
| 44 | gnl|UG|Gmr#S60866315 | cytosolic 5 -nucleotidase 3 | XP_003965851 | -1.32973 | 5.64E-22 | -0.84733 | 3.07E-12 | **-2.17706** | 4.16E-29 | -ND- | 1.51E-67 | -ND- | 4.58E-28 | -ND- | 1.51E-67 |
| 45 | gnl|UG|Gmr#S60863349 | diamine acetyltransferase 1 | ACO10033 | **2.062588** | 4.55E-38 | -1.06968 | 1.68E-25 | 0.992911 | 1.82E-16 | 0.259947 | 2.13E-18 | -ND- | 1.81E-45 | -ND- | 4.57E-37 |
| 46 | gnl|UG|Gmr#S60845504 | elongation factor 1-gamma | XP_003444636 | **2.425263** | 7.72E-44 | 0.567735 | 6.2E-32 | **2.992999** | 5.76E-72 | -1.90995 | 5.44E-90 | 1.246582 | 8.88E-46 | -0.66336 | 4.81E-46 |
| 47 | gnl|UG|Gmr_S41431673 | eno1 partial | ABE98235 | **-2.48169** | 1.5E-193 | 1.737668 | 5.7E-99 | -0.74402 | 1.3E-96 | **-2.78447** | 0 | -0.33832 | 7.2E-189 | **-3.12279** | 0 |
| 48 | gnl|UG|Gmr#S60849396 | eukaryotic translation initiation factor 3 subunit d-like | XP_003442557 | -ND- | 1.87E-14 | -ND- | 2.99E-27 | 0.992873 | 2.3E-14 | -ND- | 5.45E-64 | -ND- | 9.62E-26 | **-2.24848** | 6.15E-49 |
| 49 | gnl|UG|Gmr#S60862873 | f-actin-capping protein subunit alpha-1 | XP_003448258 | **-2.74467** | 3.6E-106 | **2.24011** | 1.03E-66 | -0.50457 | 1.28E-45 | -1.46253 | 1.1E-52 | 0.509625 | 1.38E-21 | -0.9529 | 1.08E-38 |
| 50 | gnl|UG|Gmr_S60859970 | fast myotomal muscle tropomyosin | BAC57564 | -1.59273 | 4.3E-102 | **3.000705** | 7.5E-252 | 1.407976 | 2.1E-171 | 0.996926 | 2.52E-34 | -1.33834 | 1.25E-42 | -0.34142 | 2.55E-17 |
| 51 | gnl|UG|Gmr#S60842190 | fast myotomal muscle tropomyosin | BAC44994 | -1.55207 | 8.37E-72 | **2.059576** | 1.1E-121 | 0.507506 | 2.46E-54 | -0.13237 | 7.64E-27 | 1.185193 | 9.62E-67 | 1.052822 | 6.29E-61 |
| 52 | gnl|UG|Gmr#S60844733 | fragile x mental retardation syndrome-related protein 2 | XP_003450395 | **-3.06667** | 1.15E-92 | 1.152693 | 1.53E-36 | -1.91397 | 3.93E-75 | 0.674913 | 4.96E-05 | -0.33835 | 0.002794 | 0.336565 | 0.005741 |
| 53 | gnl|UG|Gmr#S60845702 | gamma-aminobutyric acid receptor-associated protein | XP_003966959 | 1.25542 | 1.5E-20 | -ND- | 2.01E-35 | -ND- | 6.84E-17 | **2.482323** | 3.7E-100 | -ND- | 6.6E-126 | -ND- | 2.27E-49 |
| 54 | gnl|UG|Gmr#S60846355 | glycogen muscle form | ACN10567 | -ND- | 1.6E-134 | -ND- | 1.46E-52 | **-2.91387** | 7.4E-114 | 0.090038 | 4.77E-14 | -ND- | 1.11E-33 | -ND- | 2.25E-31 |
| 55 | gnl|UG|Gmr#S60865932 | guanidinoacetate n-methyltransferase | ACO14395 | **2.255345** | 1.29E-63 | 0.612114 | 1.97E-49 | **2.867459** | 2.7E-109 | -ND- | 2.4E-30 | -ND- | 7.88E-57 | 0.92161 | 2.74E-28 |
| 56 | gnl|UG|Gmr#S60862296 | heat shock cognate 70 | BAD05136 | **2.577354** | 2.36E-35 | -0.16926 | 1.71E-14 | **2.408093** | 4.28E-30 | -1.32497 | 9.37E-27 | -1.33821 | 2.05E-16 | **-2.66318** | 1.35E-38 |
| 57 | gnl|UG|Gmr#S60845698 | heat shock protein hsp 90-alpha 1-like | BAF92789 | 0.840284 | 1.13E-39 | 1.567718 | 1.33E-79 | **2.408002** | 1.3E-100 | -0.325 | 0.007663 | -0.33844 | 0.012258 | -0.66344 | 0.000261 |
| 58 | gnl|UG|Gmr_S44201072 | heavy polypeptide skeletal muscle | XP_003976385 | **-2.49095** | 0 | **2.141** | 0 | -0.34995 | 0 | -0.63535 | 0 | 0.971978 | 0 | 0.336623 | 0 |
| 59 | gnl|UG|Gmr_S41410886 | heavy polypeptide skeletal muscle | AAI55231 | **-2.12321** | 0 | 1.681609 | 0 | -0.4416 | 0 | -0.62269 | 1.1E-250 | 0.971493 | 0 | 0.348798 | 3.7E-213 |
| 60 | gnl|UG|Gmr_S55469074 | hypothetical protein | AAX30301 | 0.776131 | 4.1E-289 | **-2.19127** | 0 | -1.41514 | 3.5E-265 | **-2.07991** | 0 | -0.531 | 3.3E-291 | **-2.61091** | 0 |
| 61 | gnl|UG|Gmr#S60847604 | kelch-like protein 31 | NP_001003727 | 0.840311 | 4.99E-26 | 1.567668 | 1.14E-51 | **2.407979** | 3.85E-65 | -0.90995 | 1.14E-22 | 0.24659 | 1.15E-09 | -0.66336 | 1.58E-17 |
| 62 | gnl|UG|Gmr_S60843641 | lactate dehydrogenase-a | AAP44524 | **-3.68323** | 1.7E-237 | 1.375005 | 5.56E-92 | **-2.30822** | 2.6E-200 | **-2.58802** | 5.9E-259 | -0.07538 | 1.9E-105 | **-2.6634** | 4.3E-262 |
| 63 | gnl|UG|Gmr#S60845948 | loc100148802 protein | XP_003455232 | -ND- | 2.36E-50 | **2.737556** | 1.5E-106 | -ND- | 8.4E-129 | -ND- | 6.1E-14 | -ND- | 1.74E-32 | -ND- | 1.74E-32 |
| 64 | gnl|UG|Gmr#S60848360 | low quality protein: titin | CAG13290 | -0.9857 | 9.4E-33 | 0.278199 | 1.26E-13 | -0.7075 | 5.21E-25 | **2.13443** | 5.6E-144 | -0.71033 | 2.73E-73 | 1.424102 | 1.26E-72 |
| 65 | gnl|UG|Gmr#S41390702 | low quality protein: titin | XP_003458433 | -0.74465 | 6.27E-20 | **2.152673** | 3.34E-49 | 1.408019 | 1.86E-38 | -1.32505 | 2.27E-30 | -0.33837 | 1.94E-14 | -1.66342 | 3.53E-35 |
| 66 | gnl|UG|Gmr#S60863506 | methionine aminopeptidase 2 | XP_004083262 | 0.255306 | 0.265376 | 0.152687 | 0.323905 | 0.407993 | 0.139751 | **-2.32506** | 7.91E-37 | 0.661712 | 4.67E-15 | -1.66335 | 7.38E-31 |
| 67 | gnl|UG|Gmr#S41424332 | methyltransferase-like protein 21c-like | XP_004067197 | **-2.32966** | 7.28E-98 | -ND- | 9.09E-50 | -ND- | 1.9E-126 | -ND- | 1E-68 | -ND- | 1.54E-28 | -ND- | 1E-68 |
| 68 | gnl|UG|Gmr#S60851340 | microtubule-associated proteins 1a 1b light chain 3a | NP_999904 | **2.255223** | 2.33E-54 | -ND- | 4.87E-71 | -ND- | 1.81E-28 | -ND- | 5.24E-96 | -ND- | 5.24E-96 | -ND- | 1.7E-39 |
| 69 | gnl|UG|Gmr#S60863183 | mid1-interacting protein 1 | AEB31291 | **2.06265** | 2.11E-38 | -1.06967 | 1.02E-25 | 0.992976 | 1.32E-16 | **-2.64695** | 1.7E-101 | -0.33828 | 9.23E-43 | **-2.98523** | 2.9E-106 |
| 70 | gnl|UG|Gmr#S60844657 | middle subunit | BAM37460 | -ND- | 1.1E-284 | **-3.54776** | 4.9E-256 | -ND- | 6.7E-111 | -1.32496 | 4.3E-40 | -ND- | 3.59E-31 | -ND- | 2.68E-68 |
| 71 | gnl|UG|Gmr#S60861617 | muscleblind-like protein 1-like isoform 5 | XP_003438212 | -0.74479 | 1.36E-10 | **2.612344** | 2.88E-25 | 1.86755 | 1.35E-21 | -0.32486 | 0.126157 | 0.661558 | 0.024131 | 0.336697 | 0.095135 |
| 72 | gnl|UG|Gmr#S60848755 | musculoskeletal embryonic nuclear protein 1 | ACI67465 | -ND- | 1.19E-75 | **2.7376** | 4.7E-161 | -ND- | 7.1E-195 | -ND- | -ND- | -ND- | -ND- | -ND- | -ND- |
| 73 | gnl|UG|Gmr#S60863844 | myomesin- partial | XP_004079559 | **-3.06657** | 4.2E-161 | **2.152626** | 3.38E-78 | -0.91394 | 3.58E-85 | -0.68759 | 3.93E-17 | 0.313706 | 2.26E-08 | -0.37388 | 1.63E-10 |
| 74 | gnl|UG|Gmr_S51747641 | myomesin-1 isoform 1 | CBN82080 | **-3.552** | 1.4E-236 | **2.737579** | 6.3E-124 | -0.81442 | 4.9E-115 | -1.90999 | 1.12E-84 | 0.661671 | 9.21E-34 | -1.24832 | 1.39E-64 |
| 75 | gnl|UG|Gmr_S55473352 | myosin binding protein h | XP_003453709 | **-2.48166** | 1.8E-163 | **3.091269** | 9.2E-279 | 0.609613 | 3E-123 | -1.325 | 1.31E-94 | -0.01646 | 4.2E-39 | -1.34145 | 1.9E-95 |
| 76 | gnl|UG|Gmr_S60847006 | myosin heavy chain | BAA33452 | **-3.02952** | 0 | 1.963303 | 0 | -1.06621 | 0 | -1.16632 | 0 | 0.782653 | 0 | -0.38366 | 0 |
| 77 | gnl|UG|Gmr_S55473677 | myosin heavy chain | BAA19070 | **-2.2985** | 0 | **2.011936** | 0 | -0.28657 | 0 | -1.11067 | 0 | 0.549745 | 0 | -0.56093 | 0 |
| 78 | gnl|UG|Gmr_S41411552 | myosin heavy chain | BAA12887 | **-2.62385** | 0 | **2.100215** | 0 | -0.52363 | 0 | -0.59212 | 3.6E-193 | 0.58635 | 1.9E-190 | -0.00577 | 6.36E-78 |
| 79 | gnl|UG|Gmr_S41411187 | myosin heavy chain | BAA19070 | **-2.37857** | 0 | 1.981199 | 0 | -0.39737 | 0 | -1.98798 | 0 | 1.600237 | 0 | -0.38774 | 0 |
| 80 | gnl|UG|Gmr_S60842126 | myosin heavy chain | BAG16353 | **-2.7447** | 0 | **2.336602** | 0 | -0.4081 | 0 | -0.85456 | 7.3E-217 | 0.924673 | 9.7E-246 | 0.07011 | 1.48E-95 |
| 81 | gnl|UG|Gmr_S59565731 | myosin heavy chain | AEA36763 | **-2.8227** | 0 | **2.340301** | 0 | -0.4824 | 0 | -0.49494 | 8.44E-85 | 0.185201 | 3E-36 | -0.30974 | 2.4E-54 |
| 82 | gnl|UG|Gmr_S60842128 | myosin heavy chain | BAF75963 | **-2.86021** | 1.7E-171 | **2.889679** | 2.3E-176 | 0.029471 | 8.3E-71 | **-4.2319** | 0 | 0.66164 | 0 | **-3.57026** | 0 |
| 83 | gnl|UG|Gmr#S55479912 | myosin heavy chain | AAM22781 | -0.7447 | 1.38E-14 | 0.737645 | 2.14E-14 | -0.00706 | 1.23E-06 | **-2.13235** | 2.1E-123 | 1.983548 | 2.8E-105 | -0.14881 | 2.49E-48 |
| 84 | gnl|UG|Gmr#S60837574 | myosin heavy chain | XP_003978849 | -0.15971 | 0.002006 | -0.43229 | 8.94E-05 | -0.592 | 1.29E-06 | **-2.64697** | 1.3E-169 | 1.246633 | 5.13E-68 | -1.40034 | 3.5E-120 |
| 85 | gnl|UG|Gmr_S60867290 | myosin light chain 1 | BAA95143 | -1.06663 | 3.8E-160 | **2.211565** | 0 | 1.144939 | 7.1E-262 | -0.71204 | 1.66E-68 | 1.17065 | 2.6E-133 | 0.458614 | 9.5E-67 |
| 86 | gnl|UG|Gmr_S60867323 | myosin light chain 2 | BAB18578 | **-2.02071** | 0 | **2.58177** | 0 | 0.561058 | 0 | -1.36874 | 0 | 1.394158 | 0 | 0.025421 | 0 |
| 87 | gnl|UG|Gmr_S60868696 | myozenin 1 | XP_004077089 | -1.32968 | 3.8E-118 | **2.268174** | 2E-265 | 0.938494 | 2.5E-154 | 1.844913 | 3.6E-112 | 0.491713 | 9.75E-79 | **2.336626** | 1.1E-182 |
| 88 | gnl|UG|Gmr_S60870650 | ---NA--- |  | **-3.06662** | 2.2E-231 | **2.853105** | 1.5E-189 | -0.21351 | 1.23E-89 | **-3.86932** | 0 | **2.934646** | 0 | -0.93468 | 0 |
| 89 | gnl|UG|Gmr#S41434909 | ---NA--- |  | 0.992256 | 2.51E-60 | **-2.16925** | 8.36E-98 | -1.17699 | 1.3E-42 | -ND- | 8.2E-199 | -1.33835 | 5.7E-116 | -ND- | 1.19E-88 |
| 90 | gnl|UG|Gmr#S51747338 | ---NA--- |  | -ND- | 4.37E-92 | **2.474662** | 4.2E-188 | -ND- | 3.4E-237 | -ND- | 2.8E-126 | -ND- | 8.37E-77 | -0.6634 | 3.66E-55 |
| 91 | gnl|UG|Gmr#S44197062 | ---NA--- |  | -1.74463 | 4.13E-44 | **2.959988** | 1.3E-102 | 1.215356 | 1.63E-64 | -ND- | 5.9E-125 | -ND- | 4.18E-51 | -ND- | 5.9E-125 |
| 92 | gnl|UG|Gmr#S44201936 | ---NA--- |  | -0.74477 | 9.76E-23 | **2.152767** | 1.84E-56 | 1.407998 | 4.45E-44 | -1.90995 | 2.6E-114 | -ND- | 2.12E-65 | -ND- | 1.6E-162 |
| 93 | gnl|UG|Gmr#S60867708 | ---NA--- |  | -1.74467 | 9.72E-25 | **2.73756** | 6.35E-53 | 0.992891 | 1.47E-30 | -ND- | -ND- | -ND- | -ND- | -ND- | -ND- |
| 94 | gnl|UG|Gmr#S55468425 | ---NA--- |  | -0.74468 | 2.35E-21 | **2.152697** | 6.97E-53 | 1.408021 | 2.62E-41 | -0.32501 | 3.79E-05 | -0.33837 | 0.000109 | -0.66337 | 1.7E-08 |
| 95 | gnl|UG|Gmr#S44191132 | ---NA--- |  | 1.577204 | 1.71E-25 | **-2.16935** | 2.26E-30 | -0.59215 | 1.46E-12 | -ND- | 2.73E-83 | **-2.33837** | 1.06E-64 | -ND- | 4.15E-33 |
| 96 | gnl|UG|Gmr#S60842608 | ---NA--- |  | **-3.32952** | 1.25E-56 | 1.959946 | 3.25E-24 | -1.36958 | 2.79E-37 | 0.897462 | 6.13E-10 | 0.554703 | 5.24E-10 | 1.452165 | 1.83E-18 |
| 97 | gnl|UG|Gmr#S60847716 | ---NA--- |  | -0.15972 | 0.050747 | 0.567695 | 0.001763 | 0.407974 | 0.007795 | 1.996958 | 8.13E-44 | **-2.66018** | 4.54E-50 | -0.66323 | 3.28E-20 |
| 98 | gnl|UG|Gmr#S60842327 | nadh dehydrogenase subunit 2 | ABF21484 | **-2.32966** | 2.39E-38 | 1.737638 | 3.71E-22 | -0.59203 | 3.01E-18 | 0.674982 | 1.42E-06 | 0.246608 | 9.71E-05 | 0.921589 | 1.41E-09 |
| 99 | gnl|UG|Gmr#S55471390 | nadh dehydrogenase subunit 5 | ACF41230 | -0.74475 | 1.93E-12 | -ND- | 1.31E-15 | -ND- | 4.36E-26 | -ND- | 1.3E-136 | **-2.33833** | 7.1E-106 | -ND- | 1.12E-53 |
| 100 | gnl|UG|Gmr#S60849643 | n-alpha-acetyltransferase 50 | XP_004081108 | -ND- | 4.44E-18 | **2.152778** | 3.86E-33 | -ND- | 4.91E-44 | -0.32492 | 8.73E-07 | -ND- | 3.76E-12 | -ND- | 2.21E-15 |
| 101 | gnl|UG|Gmr_S60835498 | nebulin | CAG08263 | -1.48167 | 6.8E-134 | **2.375069** | 1.1E-290 | 0.893403 | 1.6E-161 | -1.58804 | 1.4E-268 | 1.661634 | 8.8E-294 | 0.073589 | 7.6E-115 |
| 102 | gnl|UG|Gmr_S60844359 | nebulin | CAG08263 | **-2.48166** | 3.3E-174 | **2.37507** | 1.7E-156 | -0.10659 | 2.86E-68 | **-2.64694** | 1.7E-183 | **3.831566** | 0 | 1.184629 | 2.4E-239 |
| 103 | gnl|UG|Gmr_S60870473 | nebulin | XP_003454922 | 1.255301 | 1.28E-72 | 1.240156 | 1E-118 | **2.495457** | 5.1E-177 | 0.93802 | 7.81E-71 | 0.600239 | 1.98E-74 | 1.538259 | 3.1E-143 |
| 104 | gnl|UG|Gmr_S41428103 | nebulin | XP_004081575 | 1.577232 | 8E-113 | 1.830738 | 2.9E-221 | **3.40797** | 1.4E-289 | -0.00308 | 0.201684 | 0.147063 | 0.042374 | 0.143978 | 0.044916 |
| 105 | gnl|UG|Gmr#S43507967 | nebulin | XP_004081575 | **-2.32965** | 4.58E-95 | 1.737633 | 6.21E-54 | -0.59202 | 5E-44 | 0.674972 | 1.31E-14 | 0.246593 | 4.79E-10 | 0.921565 | 4E-22 |
| 106 | gnl|UG|Gmr_S60853584 | neurobeachin | XP_003458317 | -ND- | 2.85E-29 | 1.15262 | 3.29E-33 | -ND- | 1.78E-60 | **-2.06197** | 2.2E-259 | 1.246594 | 3.6E-120 | -0.81538 | 2.3E-143 |
| 107 | gnl|UG|Gmr_S41424911 | novel protein vertebrate nebulin | XP_003454922 | 0.255246 | 3.1E-108 | **2.612131** | 4E-262 | **2.867377** | 4.8E-272 | -ND- | 3.8E-139 | -ND- | 1.45E-84 | -0.66338 | 1.09E-60 |
| 108 | gnl|UG|Gmr#S60842096 | novel protein vertebrate rna binding motif protein 33 | XP_004081342 | -0.06662 | 3.14E-30 | **-2.8473** | 2.59E-70 | **-2.91392** | 7.69E-75 | -1.06198 | 1.02E-65 | -1.92337 | 1.95E-51 | **-2.98535** | 1.9E-113 |
| 109 | gnl|UG|Gmr_S41425648 | parvalbumin | Q90YK7 | -0.25926 | 1.52E-08 | 0.667257 | 1.59E-18 | 0.407993 | 2.81E-12 | **-3.13236** | 8.4E-164 | **3.468982** | 4.3E-221 | 0.33662 | 4.7E-87 |
| 110 | gnl|UG|Gmr#S60867543 | peptidyl-prolyl cis-trans isomerase-like | AAI00003 | -1.7446 | 5.26E-30 | **2.474514** | 4.35E-57 | 0.72991 | 7.75E-29 | -ND- | 1.67E-63 | -ND- | 1.95E-26 | -ND- | 1.67E-63 |
| 111 | gnl|UG|Gmr#S55462645 | phosphoglucomutase 1 | AFL65034 | **-3.32968** | 4.7E-145 | **2.474642** | 7.93E-74 | -0.85504 | 3.61E-73 | -0.32502 | 1.16E-25 | 1.246604 | 3.53E-64 | 0.921583 | 7.16E-51 |
| 112 | gnl|UG|Gmr#S60866178 | phosphoglucomutase 1 | XP_003974425 | -ND- | 1.52E-61 | -ND- | 1.38E-24 | **-2.59209** | 5.28E-50 | -0.32499 | 1.11E-15 | **2.246584** | 6.18E-38 | 1.921598 | 4.98E-35 |
| 113 | gnl|UG|Gmr#S60867075 | predicted protein | XP_001638288 | -0.74466 | 3.39E-42 | **2.474596** | 1.7E-107 | 1.729932 | 3.4E-89 | -ND- | 5.8E-98 | -1.33839 | 1.65E-57 | -ND- | 3.69E-44 |
| 114 | gnl|UG|Gmr#S60849674 | profilin-2 | ACN10854 | -0.74497 | 0.014066 | 1.153036 | 0.00081 | 0.408064 | 0.022033 | -ND- | 1.64E-48 | **-2.33825** | 7.82E-38 | -ND- | 1.13E-19 |
| 115 | gnl|UG|Gmr#S60845375 | proteasome subunit alpha type-2 | XP_003443740 | 1.577163 | 2.24E-32 | **-2.16927** | 1.18E-38 | -0.59211 | 9.28E-16 | -ND- | 2.26E-35 | -ND- | 2.26E-35 | -ND- | 4.17E-15 |
| 116 | gnl|UG|Gmr#S60849657 | proteasome subunit alpha type-3 | ACO09997 | 1.57721 | 1.33E-39 | **-2.16922** | 2.39E-47 | -0.59201 | 4.16E-19 | -ND- | 1.7E-131 | **-2.33841** | 6.4E-102 | -ND- | 1.06E-51 |
| 117 | gnl|UG|Gmr#S60860856 | puromycin-sensitive aminopeptidase | XP_004065532 | -ND- | 4.84E-40 | **-2.65481** | 6.14E-33 | -ND- | 2.67E-16 | -ND- | 6.94E-13 | -ND- | 6.94E-13 | -ND- | 5.54E-06 |
| 118 | gnl|UG|Gmr_S41424576 | reticulon-2-like | XP_003450854 | -0.15976 | 2.19E-32 | 1.567724 | 1.36E-80 | 1.407964 | 5.26E-75 | -ND- | 0 | -ND- | 6.2E-151 | **-3.24838** | 0 |
| 119 | gnl|UG|Gmr#S60846885 | ribosomal protein l6 | XP_003974977 | 0.840139 | 2.06E-24 | 1.73769 | 4.06E-50 | **2.577829** | 4E-61 | 0.411922 | 1.53E-07 | -1.07533 | 4.15E-14 | -0.66341 | 5.24E-08 |
| 120 | gnl|UG|Gmr#S55465290 | ribosomal protein s3 | AAH41299 | **2.840203** | 1.2E-113 | 0.027148 | 3.32E-47 | **2.867351** | 1.3E-116 | 0.674981 | 3.43E-22 | 0.661645 | 5.69E-29 | 1.336626 | 1.74E-48 |
| 121 | gnl|UG|Gmr#S55479364 | ribosomal protein s6 | ACQ57903 | 1.062659 | 9.97E-52 | 1.045767 | 5.86E-79 | **2.108426** | 1.4E-122 | -ND- | 2.4E-126 | -0.6603 | 4.07E-55 | -ND- | 5.43E-77 |
| 122 | gnl|UG|Gmr#S60860857 | sarcoplasmic endoplasmic reticulum calcium atpase 1-like | P70083 | -1.32966 | 8.8E-29 | -0.84732 | 7.84E-16 | **-2.17698** | 2.8E-38 | 1.160407 | 1.77E-60 | -1.44527 | 9.62E-71 | -0.28486 | 4.05E-28 |
| 123 | gnl|UG|Gmr#S60867103 | serine incorporator 1 | ACI33992 | -ND- | 3.07E-93 | **-3.30661** | 2.52E-82 | -ND- | 6.83E-37 | -ND- | 8.96E-52 | **-2.33851** | 2.34E-40 | -ND- | 6.23E-21 |
| 124 | gnl|UG|Gmr#S60853909 | serine threonine-protein kinase 38 | NP_998621 | 0.840221 | 6.97E-31 | 1.737591 | 7.49E-64 | **2.577812** | 5.63E-78 | -ND- | 2.77E-20 | -1.3384 | 2.19E-12 | -ND- | 9.23E-10 |
| 125 | gnl|UG|Gmr#S60867985 | sorting nexin-22 | XP_003447604 | -ND- | 3.17E-24 | -ND- | 3.17E-24 | -ND- | 1.33E-10 | -ND- | 3.8E-156 | **-2.66031** | 1.1E-127 | -ND- | 7.6E-61 |
| 126 | gnl|UG|Gmr_S41427432 | titin | XP_003458433 | **-2.5927** | 0 | **2.585646** | 0 | -0.00706 | 5.3E-129 | 0.337951 | 1.88E-23 | 0.1686 | 2.76E-17 | 0.506551 | 4.72E-38 |
| 127 | gnl|UG|Gmr_S41425424 | titin | XP_003458433 | -1.74472 | 6.43E-93 | 1.85313 | 3.9E-105 | 0.108408 | 1.77E-41 | -1.325 | 1.9E-127 | **2.398593** | 2.1E-299 | 1.073591 | 1.8E-185 |
| 128 | gnl|UG|Gmr_S41425754 | titin | XP_003458433 | -1.15972 | 1.72E-28 | 1.152674 | 3.31E-28 | -0.00705 | 2.86E-12 | **2.259944** | 4.2E-212 | -0.92332 | 2E-122 | 1.336625 | 3.17E-95 |
| 129 | gnl|UG|Gmr_S60871840 | titin | XP_003458433 | -0.15976 | 5.39E-92 | **2.26818** | 8.8E-231 | **2.108425** | 2.2E-222 | 0.482334 | 2.84E-20 | 0.661638 | 1.72E-30 | 1.143971 | 9.55E-47 |
| 130 | gnl|UG|Gmr#S60866232 | titin | XP_003458433 | -1.06665 | 1.84E-42 | 1.73766 | 1.75E-86 | 0.671013 | 5.45E-46 | **2.259916** | 1.41E-96 | 0.661634 | 2.62E-79 | **2.921551** | 3.7E-172 |
| 131 | gnl|UG|Gmr#S60867704 | titin | XP_003458433 | **-3.44514** | 2.3E-171 | **2.737648** | 1E-95 | -0.7075 | 3.22E-79 | -1.49495 | 5.46E-62 | 1.121079 | 7.92E-39 | -0.37387 | 3.57E-27 |
| 132 | gnl|UG|Gmr#S60850323 | titin | XP_003458433 | **-2.15971** | 3.03E-70 | 1.96003 | 5.62E-57 | -0.19968 | 6.24E-28 | -0.0449 | 0.363594 | 0.097748 | 0.18479 | 0.052844 | 0.332096 |
| 133 | gnl|UG|Gmr_S60867017 | titin a | ABG48500 | **-2.20414** | 0 | 1.545007 | 6.6E-178 | -0.65913 | 1E-163 | -0.17302 | 3.4E-06 | 0.339712 | 2.81E-11 | 0.166692 | 1.83E-06 |
| 134 | gnl|UG|Gmr_S41426020 | titin a | ABG48500 | -1.48165 | 1.2E-128 | **2.889627** | 0 | 1.407975 | 5.2E-220 | 0.844906 | 1.38E-55 | 0.49171 | 1.53E-52 | 1.336616 | 3.6E-106 |
| 135 | gnl|UG|Gmr_S60843144 | titin a | ABG48500 | -1.91464 | 2.4E-103 | **2.612121** | 1.8E-193 | 0.697481 | 1.04E-92 | 1.375411 | 7.41E-81 | 0.131128 | 1.2E-37 | 1.506539 | 1.32E-95 |
| 136 | gnl|UG|Gmr#S41426156 | titin a | ABG48500 | -1.74474 | 1.68E-48 | **2.960061** | 4.9E-113 | 1.215323 | 5.27E-71 | -ND- | 2E-58 | -0.33835 | 1.42E-23 | -ND- | 8.44E-45 |
| 137 | gnl|UG|Gmr#S60852882 | titin a | ABG48500 | -0.74469 | 1.48E-50 | **3.322548** | 6.9E-128 | **2.577855** | 1.2E-116 | 0.790457 | 1.71E-16 | -0.13191 | 3.12E-07 | 0.65855 | 6.41E-13 |
| 138 | gnl|UG|Gmr#S41436650 | titin a | ABG48500 | **-2.32971** | 2.96E-54 | 1.152758 | 5.54E-23 | -1.17695 | 1.6E-36 | -0.32498 | 0.004112 | 0.661642 | 2.33E-05 | 0.336658 | 0.001741 |
| 139 | gnl|UG|Gmr_S41425449 | titin-like | XP_004084372 | 1.425228 | 7.13E-88 | 0.889655 | 5.5E-107 | **2.314883** | 6.3E-191 | 1.482339 | 1.81E-90 | 0.554727 | 5.45E-74 | **2.037066** | 8.6E-161 |
| 140 | gnl|UG|Gmr_S60863115 | triosephosphate isomerase | ACO09788 | **-3.15973** | 0 | -1.43229 | 2.9E-137 | **-4.59202** | 0 | 0.812497 | 1.28E-28 | -0.7978 | 3.51E-28 | 0.014697 | 2.71E-12 |
| 141 | gnl|UG|Gmr_S60868685 | tropomodulin 4 | ACO13653 | -ND- | 1.12E-67 | 0.567724 | 4.4E-44 | -ND- | 2.1E-104 | **-3.13234** | 0 | -ND- | 1.4E-207 | -ND- | 0 |
| 142 | gnl|UG|Gmr#S41459532 | tropomodulin 4 | XP_004078397 | 0.255271 | 2.93E-77 | **2.152709** | 2.5E-184 | **2.40798** | 1.2E-194 | 0.09003 | 0.190105 | -0.01643 | 0.417123 | 0.073595 | 0.246237 |
| 143 | gnl|UG|Gmr_S55468194 | tropomyosin | AEK21799 | -1.74468 | 9.35E-65 | **2.027134** | 7.18E-88 | 0.282449 | 2.66E-35 | -0.32501 | 1.4E-131 | **2.749088** | 0 | **2.424073** | 0 |
| 144 | gnl|UG|Gmr_S60842187 | tropomyosin alpha-1 chain isoform 3 | ACI34179 | **-2.23014** | 2.2E-162 | **2.354324** | 1.7E-184 | 0.124188 | 5.58E-72 | -0.32501 | 1.28E-19 | 0.732027 | 4.97E-43 | 0.407015 | 4.33E-26 |
| 145 | gnl|UG|Gmr_S60859967 | tropomyosin alpha-1 chain-like isoform 1 | XP_003440473 | **-2.74461** | 2E-143 | **4.544906** | 0 | 1.800297 | 8.5E-258 | -0.58806 | 2.26E-32 | 1.246613 | 2.26E-73 | 0.658555 | 4.95E-45 |
| 146 | gnl|UG|Gmr#S60859972 | tropomyosin alpha-3 chain isoform 8 | AAC41265 | **-2.20417** | 5.08E-77 | **2.400649** | 6.85E-94 | 0.196482 | 5.5E-37 | -0.64693 | 7.51E-35 | 1.749103 | 1.45E-86 | 1.102174 | 1.91E-63 |
| 147 | gnl|UG|Gmr_S60842632 | troponin skeletal muscle | XP_003978216 | -0.7447 | 2.1E-221 | **2.186628** | 0 | 1.441925 | 0 | 0.127493 | 7.38E-85 | 1.005593 | 6.4E-197 | 1.133086 | 1.2E-216 |
| 148 | gnl|UG|Gmr#S60861301 | tumor protein p53-inducible nuclear protein 2 | NP_001239287 | 1.840256 | 2.54E-38 | **-2.43228** | 4.42E-44 | -0.59202 | 6.92E-18 | -ND- | 1.49E-83 | -ND- | 1.49E-83 | -ND- | 1.72E-34 |
| 149 | gnl|UG|Gmr#S60842792 | u6 snrna-associated sm-like protein lsm7 | XP_004067760 | -0.55205 | 5.36E-12 | -0.03998 | 8.57E-06 | -0.59203 | 9.67E-13 | -1.173 | 1.13E-89 | -1.07536 | 1.45E-54 | **-2.24836** | 9.5E-133 |
| 150 | gnl|UG|Gmr#S60842840 | ubiquitin carboxyl-terminal hydrolase 7 isoform 1 | XP_003454989 | 0.840431 | 0.000119 | -ND- | 1.63E-08 | -ND- | 3.21E-05 | -1.90995 | 3.7E-11 | -0.33855 | 1.72E-05 | **-2.2485** | 4.8E-12 |
| 151 | gnl|UG|Gmr#S60867055 | ubiquitin carboxyl-terminal hydrolase isozyme l1 | ABW81470 | **2.255272** | 7.62E-70 | -ND- | 2.02E-91 | -ND- | 2.34E-36 | -ND- | 1.28E-50 | -ND- | 1.28E-50 | -ND- | 3.01E-21 |
| 152 | gnl|UG|Gmr#S41429396 | ubiquitin-conjugating enzyme e2 h | XP_003440412 | 0.840287 | 1.37E-13 | -1.43233 | 1.32E-19 | -0.59204 | 8.66E-09 | **2.259972** | 3.2E-189 | -ND- | 2.2E-248 | -ND- | 5.26E-97 |
| 153 | gnl|UG|Gmr#S60845300 | ubiquitin-conjugating enzyme e2 variant 1 | ACO08294 | **2.25521** | 3.22E-41 | -1.84729 | 3.64E-37 | 0.407916 | 7.21E-17 | 1.996863 | 1.21E-44 | **-2.66038** | 5.07E-51 | -0.66351 | 1.41E-20 |
|  | gnl|UG|Gmr_S60841836 | beta-actin | ABD65243 | 0.389391 | 2E-130 | -0.3803 | 2.8E-127 | 0.009089 | 1.05E-52 | 0.05634 | 0.001574 | 0.039275 | 0.004516 | 0.095615 | 2.4E-05 |
