# Supplementary material for: Substantial Downregulation of Myogenic Transcripts in Skeletal Muscle of Atlantic Cod during the Spawning Period
Source: PLoS One. 2016 Feb 4;11(2):e0148374. doi: 10.1371/journal.pone.0148374 (PMC4742245; doi:10.1371/journal.pone.0148374)
Supplement: S5 Table — (DOC) [file pone.0148374.s010.doc]

S5 Table. In silico survey of myosin heavy chain (*myh*) genes in Atlantic cod identified in Ensembl Genome Browser in release 71.

| **a. *In silico search for Atlantic cod myh gene in Ensembl Genome Browser*** | | | | | | | | | | | | | | |
| --- | --- | --- | --- | --- | --- | --- | --- | --- | --- | --- | --- | --- | --- | --- |
| myh gene | Hit in Ensembl | Gene name | Name (Projected HGNC Symbol) | Description | Transcript ID | | Location |  |  |  |  |  |  |  |
| MYH1 | No hit | ––– | ––– | ––– | ––– | | ––– |  |  |  |  |  |  |  |
| MYH2 | No hit | ––– | ––– | ––– | ––– | | ––– |  |  |  |  |  |  |  |
| MYH3 | No hit | ––– | ––– | ––– | ––– | | ––– |  |  |  |  |  |  |  |
| MYH4 | No hit | ––– | ––– | ––– | ––– | | ––– |  |  |  |  |  |  |  |
| MYH5 | No hit | ––– | ––– | ––– | ––– | | ––– |  |  |  |  |  |  |  |
| MYH6 | 1 | Myh6 | B3SVJ6_GADMO | Cardiac muscle myosin heavy chain 6 alpha | ENSGMOT00000022229 | | GeneScaffold_2769:37178-42967:-1 | | | |  |  |  |  |
| MYH7 | No hit | ––– | ––– | ––– | ––– | | ––– |  |  |  |  |  |  |  |
| MYH8 | No hit | ––– | ––– | ––– | ––– | | ––– |  |  |  |  |  |  |  |
| MYH9 | 2 | Myh9 (1 of 2) | MYH9 (1 of 2) | myosin, heavy chain 9, non-muscle | ENSGMOT00000012353 | | GeneScaffold_1335:35770-67772:-1 | | | |  |  |  |  |
|  |  | Myh9 (2 of 2) | MYH9 (2 of 2) | | ENSGMOT00000015399 | | GeneScaffold_3791:145323-172903:-1 | | | |  |  |  |  |
| MYH10 | 2 | Myh10 (1 of 2) | MYH10 (1 of 2) | myosin, heavy chain 10, non-muscle | ENSGMOT00000015192 | | GeneScaffold_3791:250-85058:1 | | | |  |  |  |  |
|  |  | Myh10 (2 of 2) | MYH10 (2 of 2) | | ENSGMOT00000004197 | | GeneScaffold_1589:96-106731:-1 | | | |  |  |  |  |
| MYH11 | 2 | Myh11 (1 of 2) | MYH11 (1 of 2) | myosin, heavy chain 11, smooth muscle | ENSGMOT00000006246 | | GeneScaffold_3945:145210-187352:-1 | | | |  |  |  |  |
|  |  | Myh11 (2 of 2) | MYH11 (2 of 2) | | ENSGMOT00000010575 | | GeneScaffold_3135:58223-84216:1 | | | |  |  |  |  |
| MYH12 | 1 | Myh12 | MYO5A | myosin VA (heavy chain 12, myoxin) | ENSGMOT00000017347 | | GeneScaffold_3426:122-43499:-1 | | | |  |  |  |  |
| MYH13 | 6 | Myh13 (1 of 6) | MYH13 (1 of 6) | myosin, heavy chain 13, skeletal muscle | ENSGMOT00000018065 | | GeneScaffold_3580:30698-47836:1 | | | |  |  |  |  |
|  |  | Myh13 (2 of 6) | MYH13 (2 of 6) | | ENSGMOT00000017982 | | GeneScaffold_3580:58-11235:1 | | |  |  |  |  |  |
|  |  | Myh13 (3 of 6) | MYH13 (3 of 6) | | ENSGMOT00000010416 | | GeneScaffold_355:99107-116066:-1 | | | |  |  |  |  |
|  |  | Myh13 (4 of 6) | MYH13 (4 of 6) | | ENSGMOT00000017719 | | GeneScaffold_722:1003-24714:1 | | | |  |  |  |  |
|  |  | Myh13 (5 of 6) | MYH13 (5 of 6) | | ENSGMOT00000010469 | | GeneScaffold_355:127199-138846:1 | | | |  |  |  |  |
|  |  | Myh13 (6 of 6) | MYH13 (6 of 6) | | ENSGMOT00000010750 | | GeneScaffold_575:20577-39368:-1 | | | |  |  |  |  |
| MYH14 | 2 | Myh14 (1 of 2) | MYH14 | myosin, heavy chain 14, non-muscle | ENSGMOT00000006152 | | GeneScaffold_1960:840157-877374:-1 | | | |  |  |  |  |
|  |  | Myh14 (2 of 2) | MYH7B | myosin, heavy chain 7B, cardiac muscle, beta | ENSGMOT00000013958 | | GeneScaffold_92:21379-49726:-1 | | | |  |  |  |  |
|  |  |  |  |  |  | |  |  |  |  |  |  |  |  |
| **b. *Ensembl transcripts found in the fast skeletal muscle cDNA library in Atlantic cod*** | | | | | | | | | | | | | | |
| Transcript ID | Blastx search | Gene name | Name (Projected HGNC Symbol) | Description | Transcript ID | Location | |  |  |  |  |  |  |  |
| ENSGMOT00000012320 | myh | ––– | ––– | Novel protein coding | ENSGMOT00000012320 | GeneScaffold_4616:5649-17951:1 | | | | |  |  |  |  |
| ENSGMOT00000018101 | myh | ––– | ––– | Novel protein coding | ENSGMOT00000018101 | GeneScaffold_3580:69404-77491:1 | | | | |  |  |  |  |
| ENSGMOT00000007468 | myh | ––– | ––– | Novel protein coding | ENSGMOT00000007468 | GeneScaffold_3738:52448-64291:1 | | | | |  |  |  |  |
| ENSGMOT00000017982 | myh | Myh13 (2 of 6) | MYH13 (2 of 6) | myosin, heavy chain 13, skeletal muscle | ENSGMOT00000017982 | GeneScaffold_3580:58-11235:1 | | | |  |  |  |  |  |
| ENSGMOT00000018065 | myh | Myh13 (1 of 6) | MYH13 (1 of 6) | myosin, heavy chain 13, skeletal muscle | ENSGMOT00000018065 | GeneScaffold_3580:30698-47836:1 | | | | |  |  |  |  |
|  |  |  |  |  |  |  | |  |  |  |  |  |  |  |
| **c. *Ensembl blastn search of UniGene transcripts found in the fast skeletal muscle cDNA library in Atlantic cod*** | | | | | | | | | | | | | | |
| UniGene_uniq | Blastx search | Gene name | Name (Projected HGNC Symbol) | Description | Transcript ID | Location | | Start | End | Ori | Score | E-val | %ID | Length |
| gnl|UG|Gmr_S60847006 | myh | ––– | ––– | ––– | NA | contig267509: 1-542 | | ––– | ––– | ––– | 1282 | 3.6E-219 | 100 | 256 |
| gnl|UG|Gmr_S55473677 | myh | ––– | ––– | ––– | NA | GeneScaffold_4616: 25,676-29,780 | | 27676 | 27780 | + | 527 | 7E-85 | 100 | 105 |
| gnl|UG|Gmr_S41411552 | myh | Myh13 (2 of 6) | MYH13 (2 of 6) | myosin, heavy chain 13, skeletal muscle | ENSGMOT00000017982 | GeneScaffold_3580:58-11235:1 | | 2131 | 2179 | + | 245 | 8.6E-35 | 100 | 49 |
| gnl|UG|Gmr_S41411187 | myh | ––– | ––– | ––– | low quality alignment in Ensembl | | | ––– | ––– | ––– | ––– | ––– | ––– | ––– |
| gnl|UG|Gmr_S60842126 | myh | Myh13 (2 of 6) | MYH13 (2 of 6) | myosin, heavy chain 13, skeletal muscle | ENSGMOT00000017982 | GeneScaffold_3580:58-11235:1 | | 9956 | 10159 | + | 1030 | 2.5E-174 | 100 | 204 |
| gnl|UG|Gmr_S59565731 | myh | ––– | ––– | ––– | NA | contig745186: 1-150 | | ––– | ––– | ––– | 709 | 2.8E-117 | 100 | 141 |
| gnl|UG|Gmr_S60842128 | myh | Myh13 (1 of 6) | MYH13 (1 of 6) | myosin, heavy chain 13, skeletal muscle | ENSGMOT00000018065 | GeneScaffold_3580:30698-47836:1 | | 47571 | 47702 | + | 667 | 7.1E-110 | 100 | 132 |
| gnl|UG|Gmr#S55479912 | myh | ––– | ––– | Novel protein coding | ENSGMOT00000012276 | GeneScaffold_1267:4270-17076:1 | | 16077 | 16202 | + | 633 | 7.6E-104 | 100 | 126 |
| gnl|UG|Gmr#S60837574 | myh | ––– | ––– | Novel protein coding | ENSGMOT00000012276 | GeneScaffold_1267:4270-17076:1 | | 4285 | 4476 | + | 972 | 5.9E-164 | 100 | 192 |
