# Supplementary material for: Substantial Downregulation of Myogenic Transcripts in Skeletal Muscle of Atlantic Cod during the Spawning Period
Source: PLoS One. 2016 Feb 4;11(2):e0148374. doi: 10.1371/journal.pone.0148374 (PMC4742245; doi:10.1371/journal.pone.0148374)
Supplement: S6 Table — (DOC) [file pone.0148374.s011.doc]

S6 Table. Teleost myosin heavy chain 13 (*myh13*) genes found in Ensembl Genome Browser in release 71 (April 2013).

| **Species** | **Gene** | **Transcript ID** |
| --- | --- | --- |
| pufferfish | MYH13 (1 of 7) | ENSTNIT00000004011 |
|  | MYH13 (2 of 7) | ENSTNIT00000009727 |
|  | MYH13 (3 of 7) | ENSTNIT00000017955 |
|  | MYH13 (4 of 7) | ENSTNIT00000006630 |
|  | MYH13 (5 of 7) | ENSTNIT00000017959 |
|  | MYH13 (6 of 7) | ENSTNIT00000009992 |
|  | MYH13 (7 of 7) | ENSTNIT00000001818 |
| Atlantic cod | MYH13 (1 of 6) | ENSGMOT00000018065 |
|  | MYH13 (2 of 6) | ENSGMOT00000017982 |
|  | MYH13 (3 of 6) | ENSGMOT00000010416 |
|  | MYH13 (4 of 6) | ENSGMOT00000017719 |
|  | MYH13 (5 of 6) | ENSGMOT00000010469 |
|  | MYH13 (6 of 6) | ENSGMOT00000010868 |
| zebrafish | MYH13 (6 of 9) | ENSDART00000051360 |
|  | MYH13 (7 of 9) | ENSDART00000123920 |
|  | MYH13 (8 of 9) | ENSDART00000075210 |
| medaka | MYH13 (1 of 10) | ENSORLT00000007920 |
|  | MYH13 (10 of 10) | ENSORLT00000002524 |
|  | MYH13 (3 of 10) | ENSORLT00000007733 |
|  | MYH13 (4 of 10) | ENSORLT00000025397 |
|  | MYH13 (5 of 10) | ENSORLT00000007821 |
|  | MYH13 (6 of 10) | ENSORLT00000016370 |
|  | MYH13 (7 of 10) | ENSORLT00000024947 |
|  | MYH13 (8 of 10) | ENSORLT00000023167 |
| stickleback | MYH13 (1 of 8) | ENSGACT00000013267 |
|  | MYH13 (2 of 8) | ENSGACT00000003867 |
|  | MYH13 (3 of 8) | ENSGACT00000003840 |
|  | MYH13 (4 of 8) | ENSGACT00000013245 |
|  | MYH13 (5 of 8) | ENSGACT00000003927 |
|  | MYH13 (6 of 8) | ENSGACT00000003951 |
|  | MYH13 (7 of 8) | ENSGACT00000001991 |
|  | MYH13 (8 of 8) | ENSGACT00000002005 |
