# Supplementary material for: Substantial Downregulation of Myogenic Transcripts in Skeletal Muscle of Atlantic Cod during the Spawning Period
Source: PLoS One. 2016 Feb 4;11(2):e0148374. doi: 10.1371/journal.pone.0148374 (PMC4742245; doi:10.1371/journal.pone.0148374)
Supplement: S7 Table — (DOC) [file pone.0148374.s012.doc]

S7 Table. Zebrafish myosin heavy chain (*myh*) genes found in NCBI (June 2013).

| **Type** | **Gene** | **Sequence description in NCBI** | **Length** | **Accession no.** |
| --- | --- | --- | --- | --- |
| Fast | myhz1.1 | [Danio rerio myosin, heavy polypeptide 1.1, skeletal muscle (myhz1.1), transcript variant 2, mRNA](http://www.ncbi.nlm.nih.gov/nuccore/NM_001130666.2) | 5,903 bp | NM_001130666 |
|  | myhz1.1 | [Danio rerio myosin, heavy polypeptide 1.1, skeletal muscle (myhz1.1), transcript variant 1, mRNA](http://www.ncbi.nlm.nih.gov/nuccore/NM_001115089.2) | 6,010 bp | NM_001115089 |
|  | myhz1.2 | [Danio rerio myosin, heavy polypeptide 1.2, skeletal muscle (myhz1.2), mRNA](http://www.ncbi.nlm.nih.gov/nuccore/NM_001161446.1) | 5,975 bp | NM_001161446 |
|  | myhz2 | [Danio rerio myosin, heavy polypeptide 2, fast muscle specific (myhz2), mRNA](http://www.ncbi.nlm.nih.gov/nuccore/NM_152982.2) | 5,991 bp | NM_152982 |
|  | myhc4 | Danio rerio fast myosin heavy chain 4 (myhc4) mRNA, complete cds | 5913 bp | AY921650 |
| Non-muscle | myh9a | [Danio rerio myosin, heavy polypeptide 9a, non-muscle (myh9a), mRNA](http://www.ncbi.nlm.nih.gov/nuccore/NM_001098177.2) | 6,842 bp | NM_001098177 |
|  | myh9b | [PREDICTED: Danio rerio myosin, heavy polypeptide 9b, non-muscle (myh9b), partial mRNA](http://www.ncbi.nlm.nih.gov/nuccore/XM_001920063.3) | 5,264 bp | XM_001920063 |
|  | myh10 | [PREDICTED: Danio rerio myosin, heavy polypeptide 10, non-muscle (myh10), mRNA](http://www.ncbi.nlm.nih.gov/nuccore/XM_677954.5) | 7,412 bp | XM_677954 |
| Smooth | myh11a | [Danio rerio myosin, heavy polypeptide 11, smooth muscle a (myh11a), mRNA](http://www.ncbi.nlm.nih.gov/nuccore/NM_001024448.1) | 7109 bp | NM_001024448 |
| Slow | smyhc1 | [Danio rerio slow myosin heavy chain 1 (smyhc1), mRNA](http://www.ncbi.nlm.nih.gov/nuccore/NM_001020507.1) | 5,905 bp | NM_001020507 |
|  | smyhc2 | [Danio rerio slow myosin heavy chain 2 (smyhc2), mRNA](http://www.ncbi.nlm.nih.gov/nuccore/NM_001102626.2) | 5,896 bp | NM_001102626 |
|  | smyhc3 | [Danio rerio slow myosin heavy chain 3 (smyhc3), mRNA](http://www.ncbi.nlm.nih.gov/nuccore/NM_001136523.1) | 5,985 bp | NM_001136523 |
| Cardiac | vmhc | [Danio rerio ventricular myosin heavy chain (vmhc), mRNA](http://www.ncbi.nlm.nih.gov/nuccore/NM_001112733.1) | 5,991 bp | NM_001112733 |
|  | amhc | [Danio rerio atrial myosin heavy chain mRNA, complete cds](http://www.ncbi.nlm.nih.gov/nuccore/AY138982.1) | 5,946 bp | AY138982 |
|  | myh6 | [Danio rerio myosin, heavy polypeptide 6, cardiac muscle, alpha (myh6), mRNA](http://www.ncbi.nlm.nih.gov/nuccore/NM_198823.1) | 5,946 bp | NM_198823 |
