# Supplementary material for: Substantial Downregulation of Myogenic Transcripts in Skeletal Muscle of Atlantic Cod during the Spawning Period
Source: PLoS One. 2016 Feb 4;11(2):e0148374. doi: 10.1371/journal.pone.0148374 (PMC4742245; doi:10.1371/journal.pone.0148374)
Supplement: S8 Table — (DOCX) [file pone.0148374.s013.docx]

| **Gene** | **Contig title/Acc. number** | **Contig length** | **Frame** |  | **Primer sequence (5'>3')** | | **Amplicon (bp)** | **Efficiency (%)** | **R^2^** |  |
| --- | --- | --- | --- | --- | --- | --- | --- | --- | --- | --- |
| myosin heavy chain (*myh*) | gnl\|UG\|Gmr#S60847006 | 738 | 1 |  | Forward primer | ATGTAACGGTGTGCTGGAGG | 187 | 99.0 | 0.995 |  |
|  |  |  |  |  | Reverse primer | GTACTCGTCGTGAGGCACAT |  |  |  |  |
| myosin light chain 2 (*myl2*) | gnl\|UG\|Gmr#S60867323 | 1 345 | 2 |  | Forward primer | GGACGTGCTGGCCTCTATGG | 135 | 73.8 | 0.993 |  |
|  |  |  |  |  | Reverse primer | GGATCCGCACCCTTCAGCTT |  |  |  |  |
| actin, alpha cardiac muscle 1 (*actc1*) | gnl\|UG\|Gmr#S60864027 | 1 609 | 3 |  | Forward primer | CTTCCCTGTCCACCTTCCAG | 122 | 106.1 | 0.984 |  |
|  |  |  |  |  | Reverse primer | ACGGAGACGACGATGGAGAA |  |  |  |  |
| actin, alpha skeletal muscle (*acta*) | gnl\|UG\|Gmr#S44200679 | 602 | -2 |  | Forward primer | TGTTCACAGTTCGTTCTCCGA | 200 | 90.3 | 0.998 |  |
|  |  |  |  |  | Reverse primer | TCGTCTCCGTCGTCATCATC |  |  |  |  |
| novel sal-like protein (*sall*) | gnl\|UG\|Gmr#S60854098 | 995 | 3 |  | Forward primer | GTCAGAGAGAGATCCGCTTGG | 125 | 92.0 | 0.931 |  |
|  |  |  |  |  | Reverse primer | TGTGCCTGCTGAAAGAGACC |  |  |  |  |
| *arp* | EX741373 | n/a |  |  | Forward primer | TGATCCTCCACGACGATGAG | 113 | 88.2 | 0.993 |  |
|  |  |  |  |  | Reverse primer | CAGGGCCTTGGCGAAGA |  |  |  |  |
| *ubi* | EX735613 | n/a |  |  | Forward primer | GGCCGCAAAGATGCAGAT | 69 | 99.8 | 0.985 |  |
|  |  |  |  |  | Reverse primer | CTGGGCTCGACCTCAAGAGT |  |  |  |  |
| *eef1a* | EX721840 | n/a |  |  | Forward primer | CACTGAGGTGAAGTCCGTTG | 142 | 95.4 | 0.993 |  |
|  |  |  |  |  | Reverse primer | GGGGTCGTTCTTGCTGTCT |  |  |  |  |

S8 Table. Gene name, contig title/GenBank accession number, contig length, frame, primer sequences (5’ to 3’), amplicon size (bp), PCR efficiency (%) and r2 value of the DEGs and endogenous reference genes in Atlantic cod.
